# Supplementary material for: Conducting the RBD of SARS-CoV-2 Omicron Variant with Phytoconstituents from Euphorbia dendroides to Repudiate the Binding of Spike Glycoprotein Using Computational Molecular Search and Simulation Approach
Source: Molecules. 2022 May 4;27(9):2929. doi: 10.3390/molecules27092929 (PMC9099834; doi:10.3390/molecules27092929)
Supplement: Supplementary file 1 [file molecules-27-02929-s001.zip › molecules-1688603-supplementary.pdf]

Supplementary material

Conducting the RBD of SARS-COV-2 Omicron Variant with Phytoconstituents from *Euphorbia dendroides* to Repudiate the Binding of Spike Glycoprotein Using Computational Molecular Search and Simulation Approach

Table S1. Compound name, 2D-chemical structure, and IUPAC name of the seventeen studied compounds.

Compound No.	2D-Chemical Structure	Compound Name
**1**	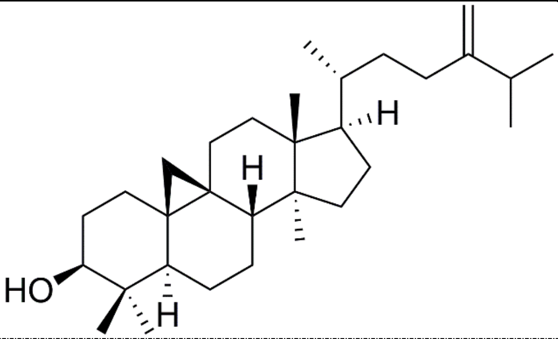	24-methylene cycloartan-3*β*-ol
**2**	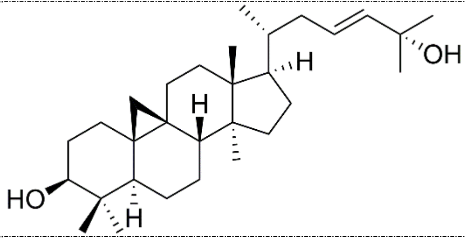	Cycloart-23-ene-3*β*,25-diol
**3**	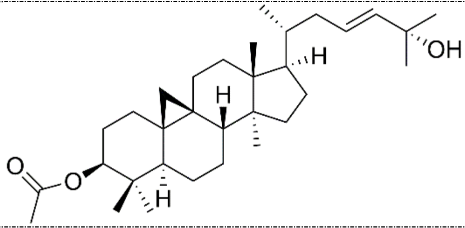	Cycloart-23-ene-3*β*,25-diol monoacetate
**4**	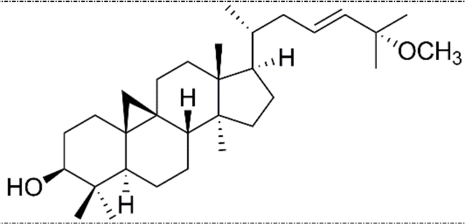	3*β*-hydroxy-cycloart-23-ene-25 methyl ether
**5**	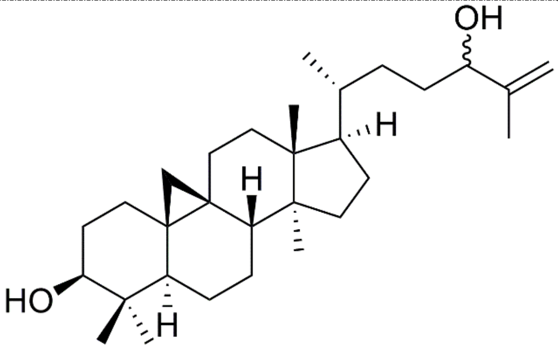	24 *R*/*S*-3*β*-hydroxy-25-methylene cycloartan-24-ol
**6**	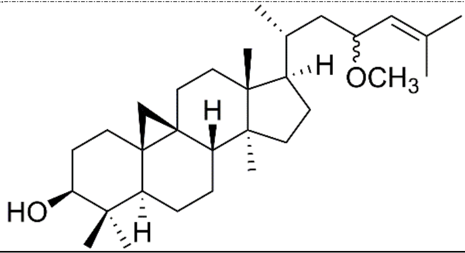	23 *R*/*S*-3*β* -hydroxycycloart-24-ene-23-methyl ether

**7**	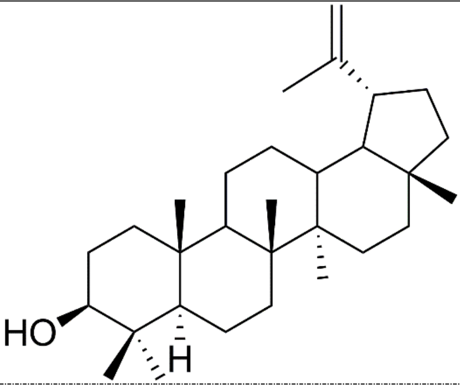	Lupeol
**8**	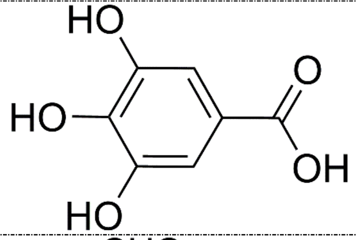	Gallic acid
**9**	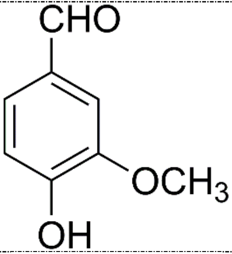	Vanillin
**10**	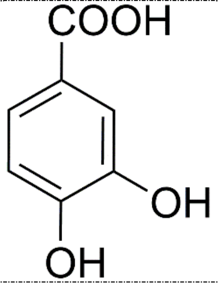	Protocatechuic acid
**11**	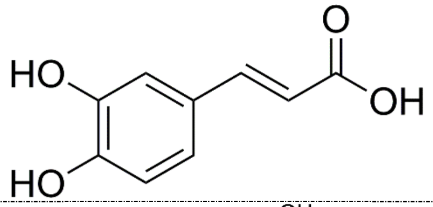	*trans*-caffeic acid
**12**	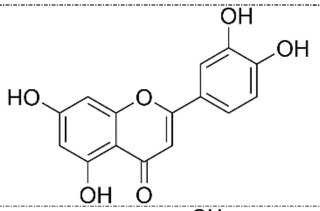	Luteolin
**13**	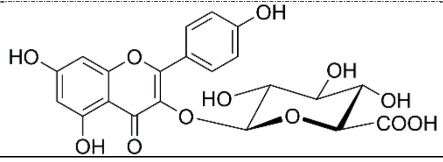	Kaempferol-3-*O*-*β*-D-glucuronopyranoside

**14**	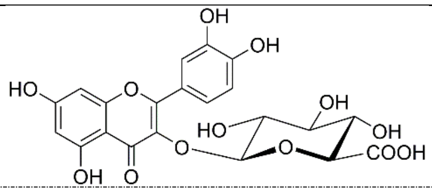	Quercetin-3-*O*-*β*-D-glucuronopyranoside
**15**	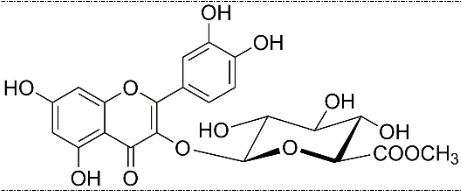	Quercetin-3-*O*-glucuronoide 6"-*O*-methyl ester
**16**	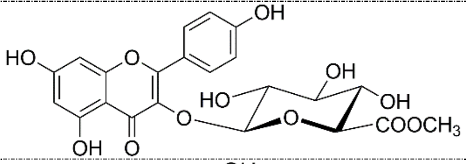	Kampferol-3-*O*-glucuronoide 6"-*O*-methyl ester
**17**	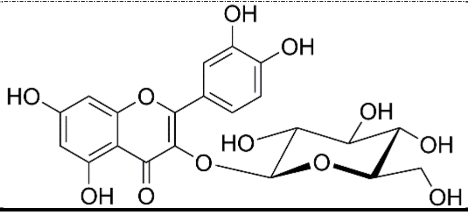	Quercetin-3-*O*-*β*-D-glucopyranoside

Table S2. Calculated docking scores (in kcal/mol) for the SARS-COV-2 Omicron drug candidates.

Compound No.	Docking Score (kcal/mol)
**1**	−4.4
**2**	−5.7
**3**	−6.4
**4**	−5.3
**5**	−4.7
**6**	−6.1
**7**	−5.0
**8**	−6.8
**9**	−7.2
**10**	−7.6
**11**	−7.7
**12**	−7.9
**13**	−8.1
**14**	−8.7
**15**	−8.8
**16**	−8.4
**17**	−8.3

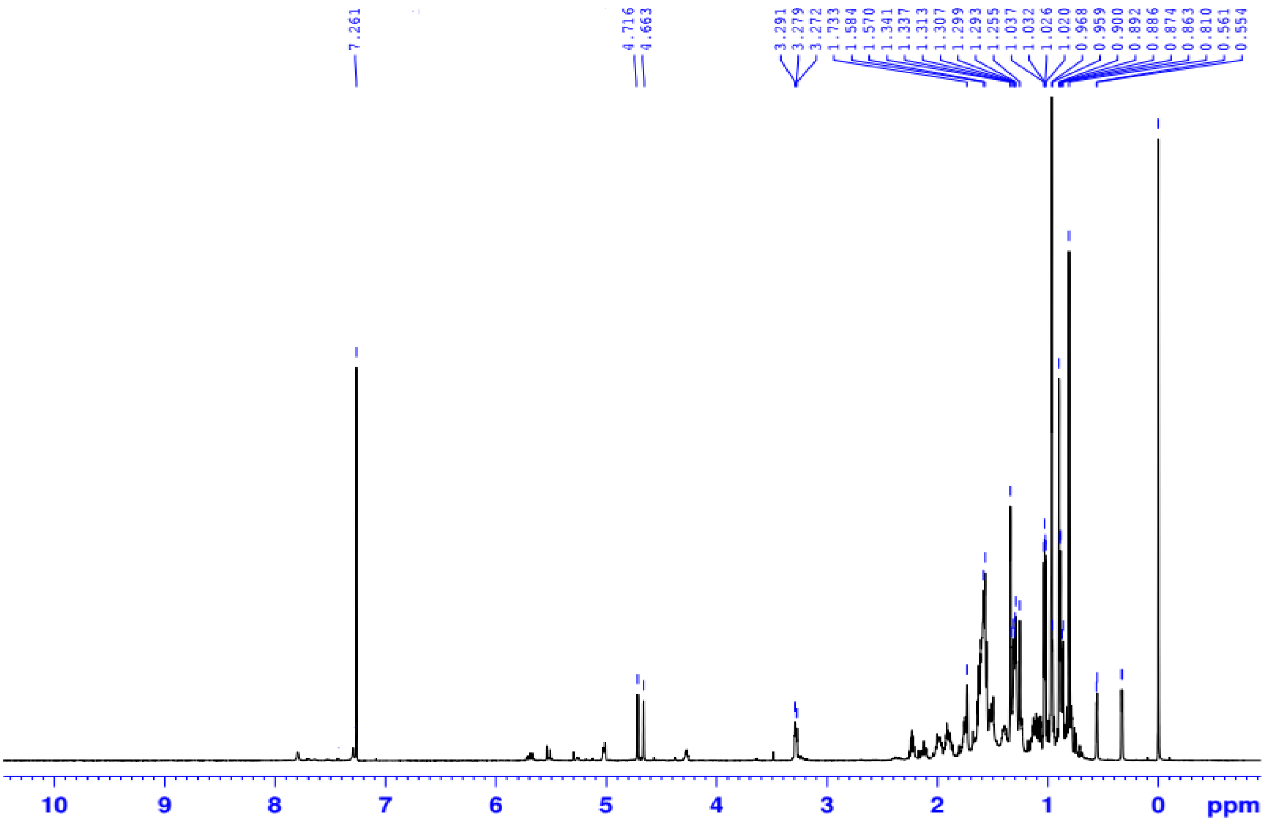

Figure S1. ^{1}H NMR spectrum (600 MHz, $CDCl_3$) of compound 1

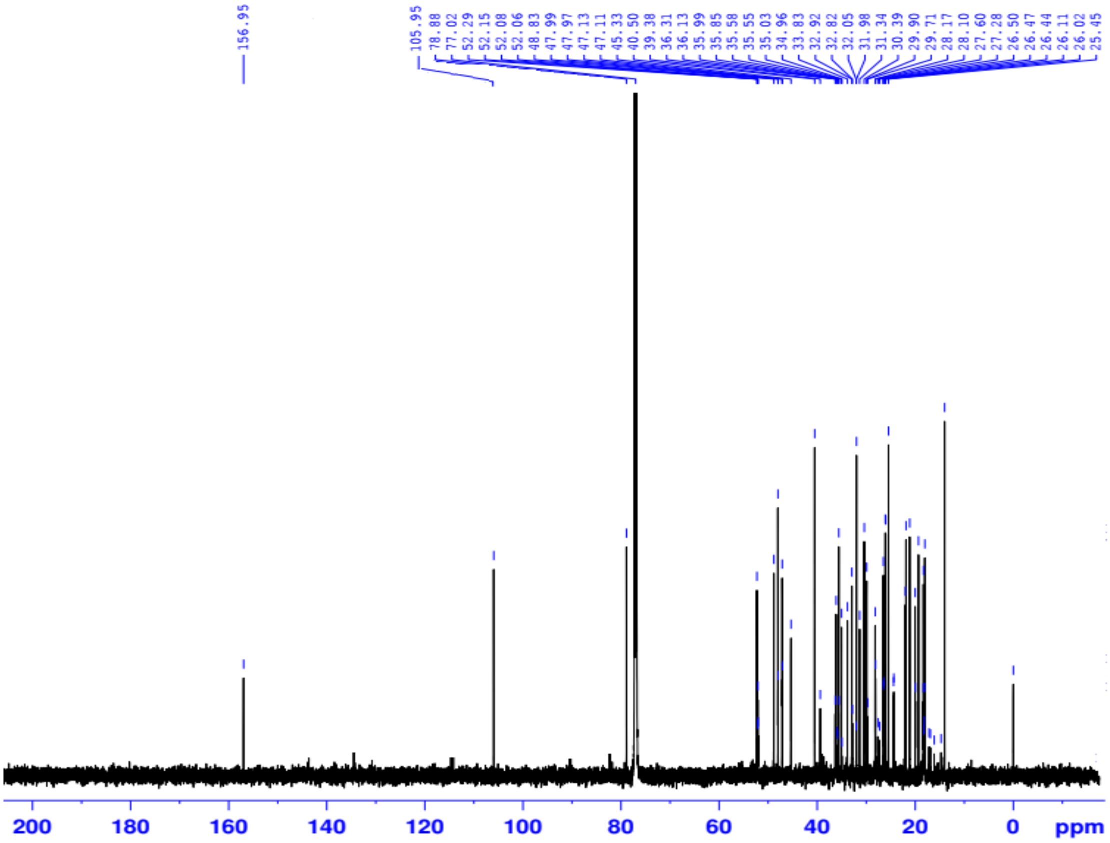

Figure S2. ^{13}C NMR spectrum (150 MHz, $CDCl_3$) of compound 1

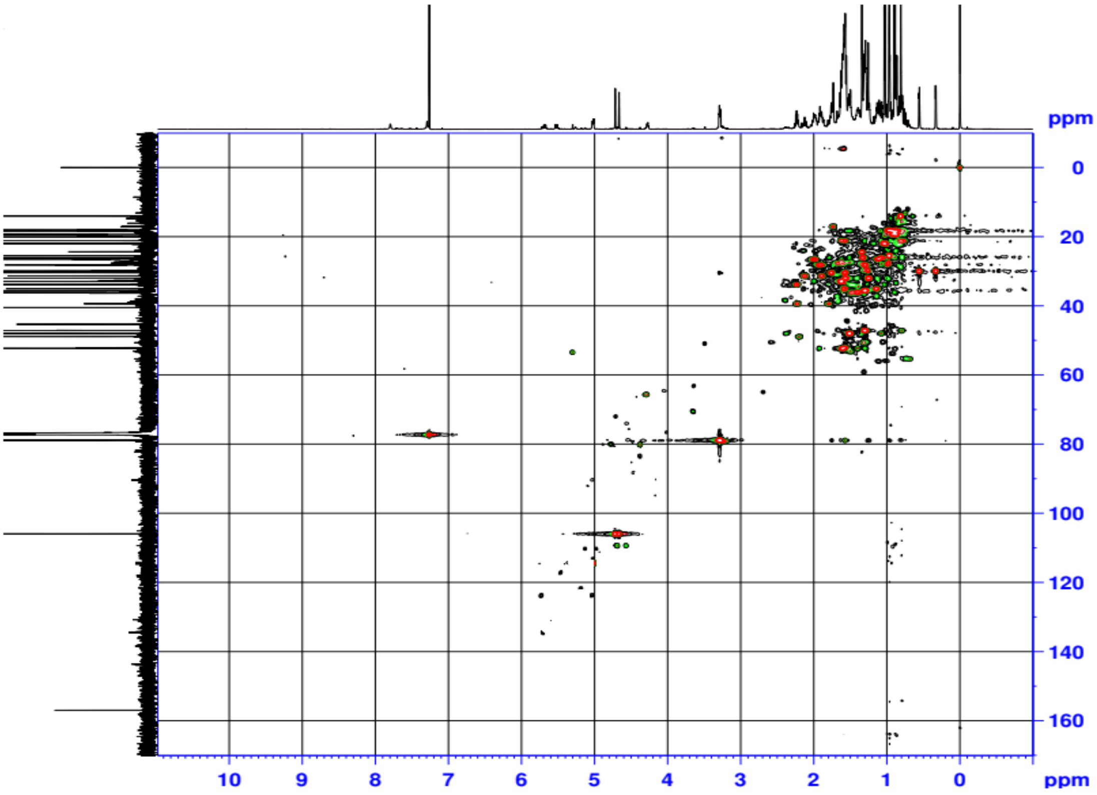

Figure S3. HSQC spectrum ($CDCl_3$) of compound 1

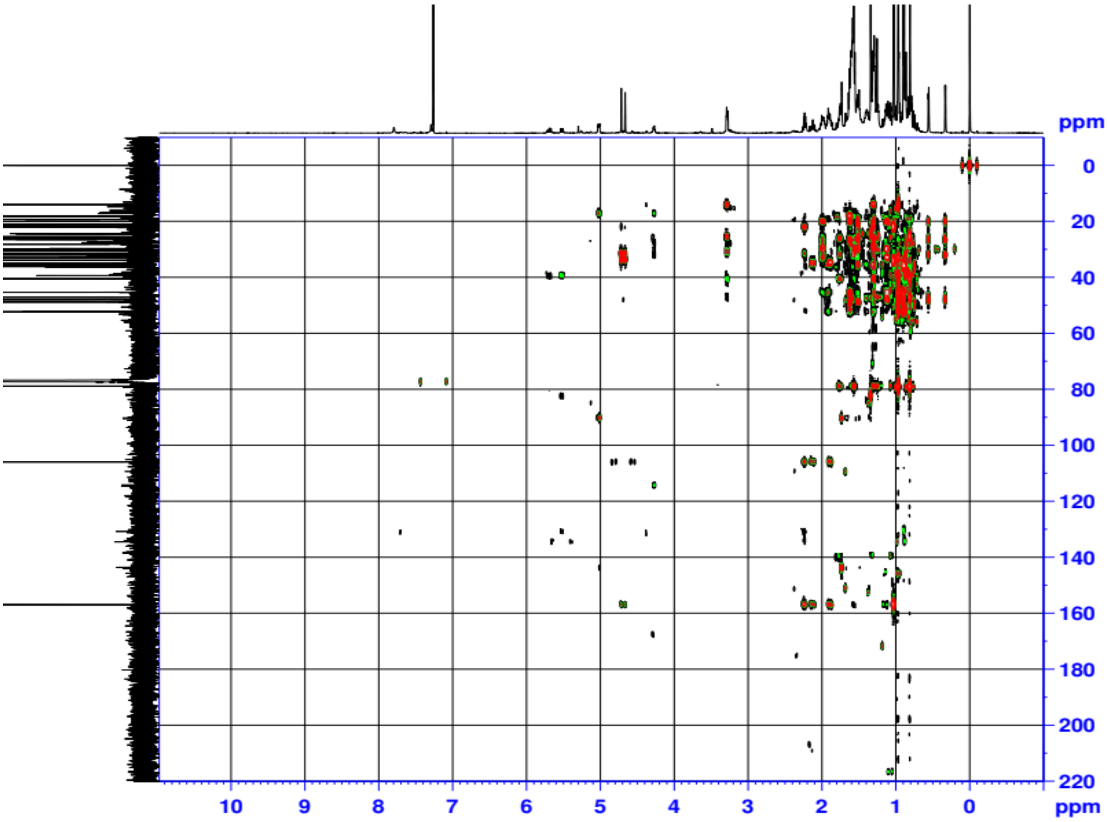

Figure S4. HMBC spectrum ($CDCl_3$) of compound 1

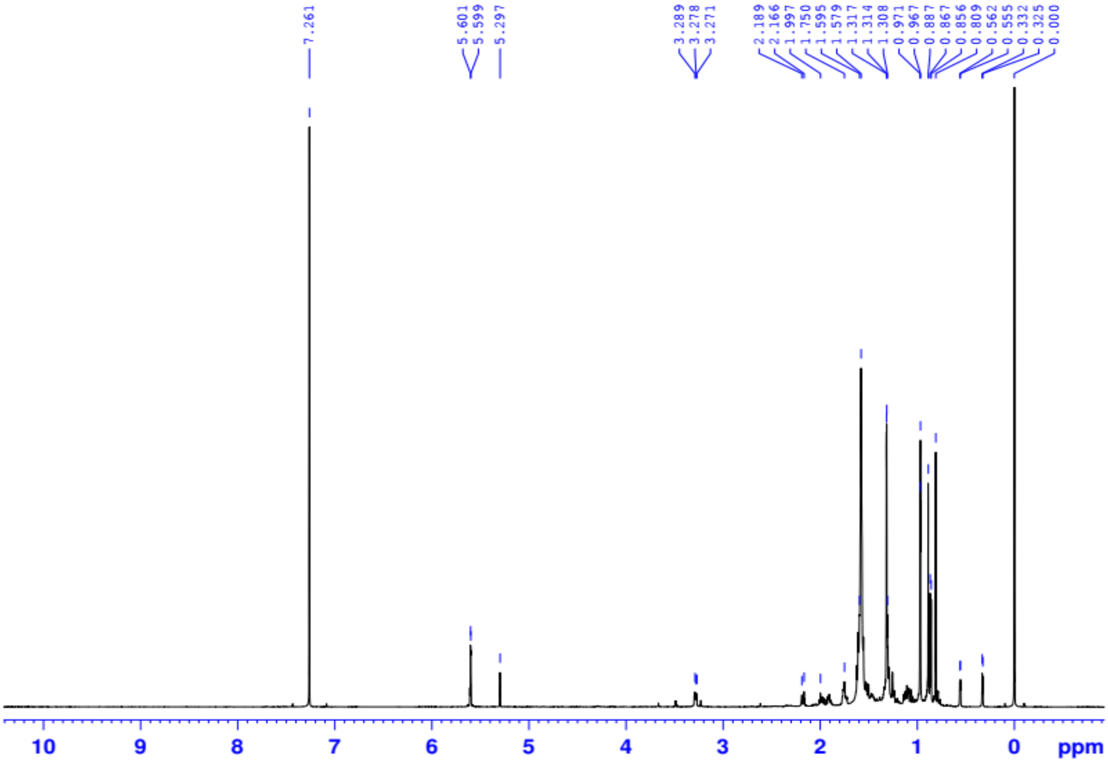

Figure S5. ^{1}H NMR spectrum (600 MHz, $CDCl_3$) of compound 2

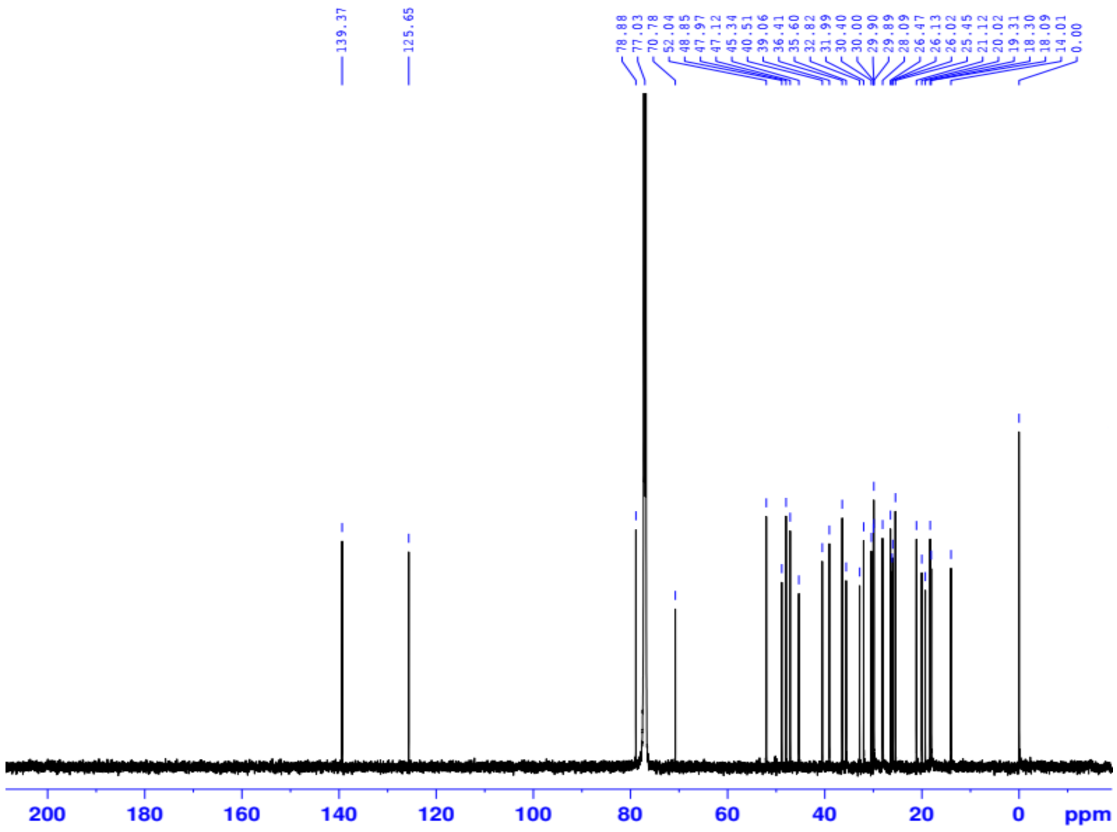

Figure S6. ^{13}C NMR spectrum (150 MHz, $CDCl_3$) of compound 2

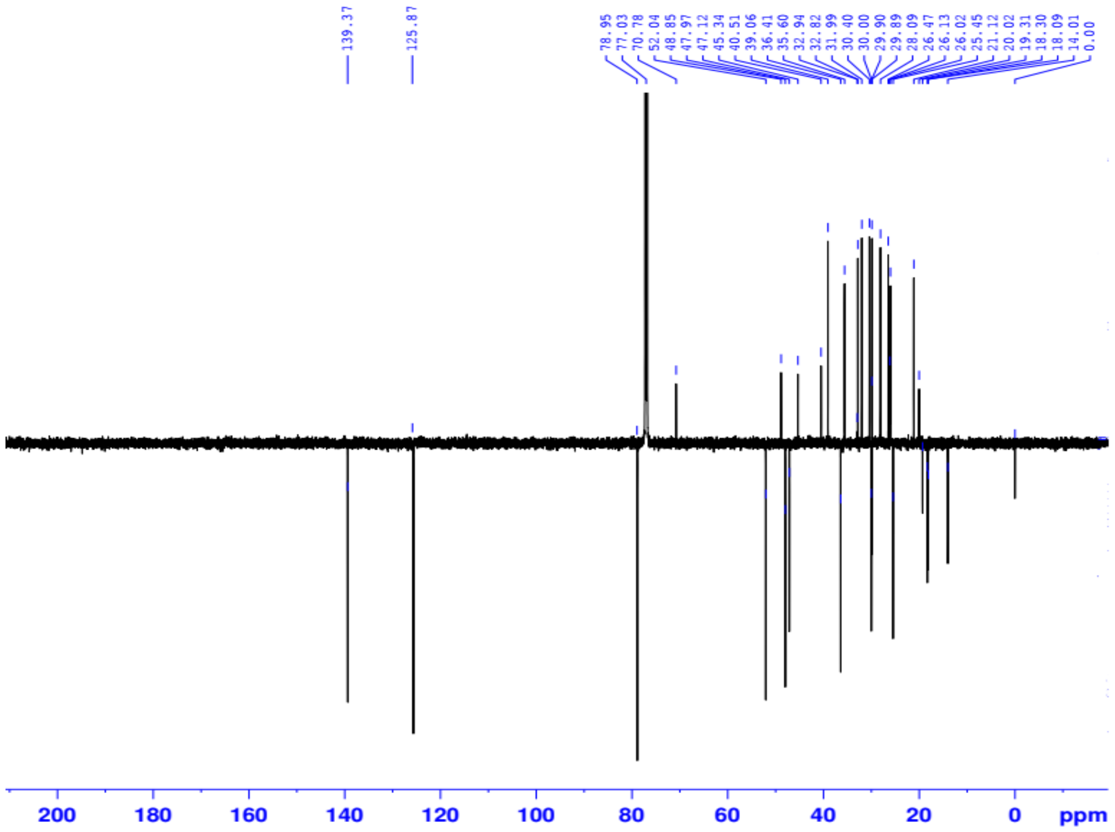

Figure S7. APT spectrum (150 MHz, $CDCl_3$) of compound 2

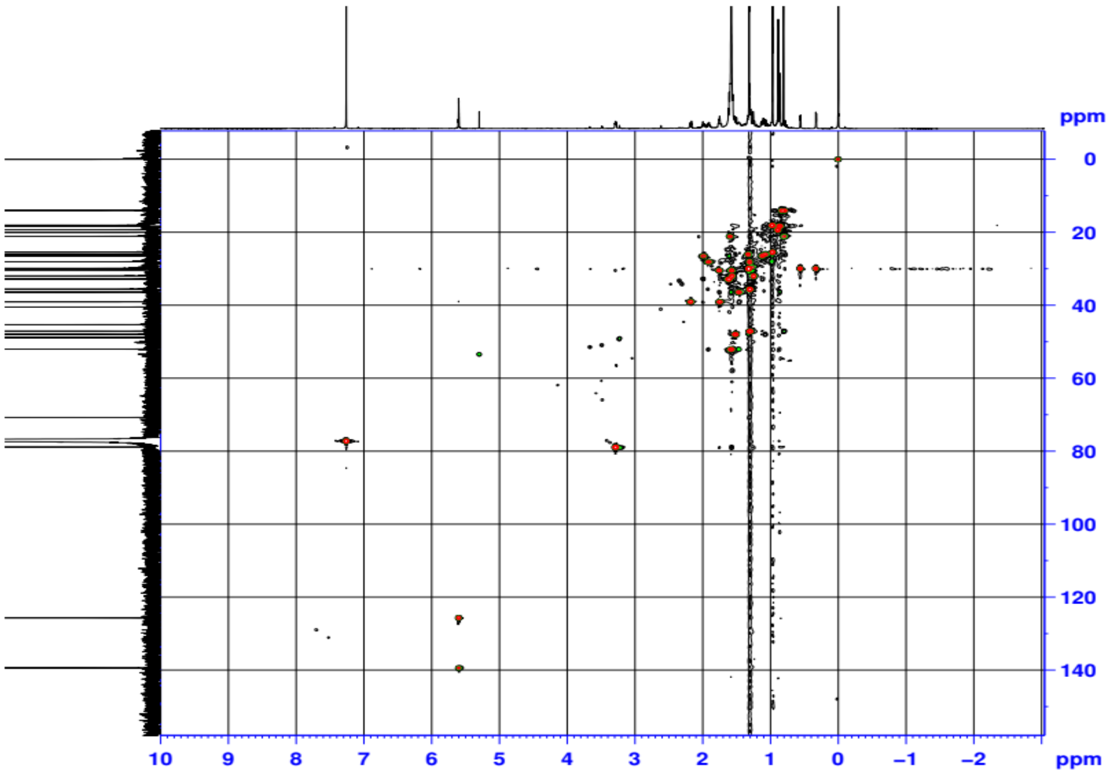

Figure S8. HSQC spectrum ($CDCl_3$) of compound 2

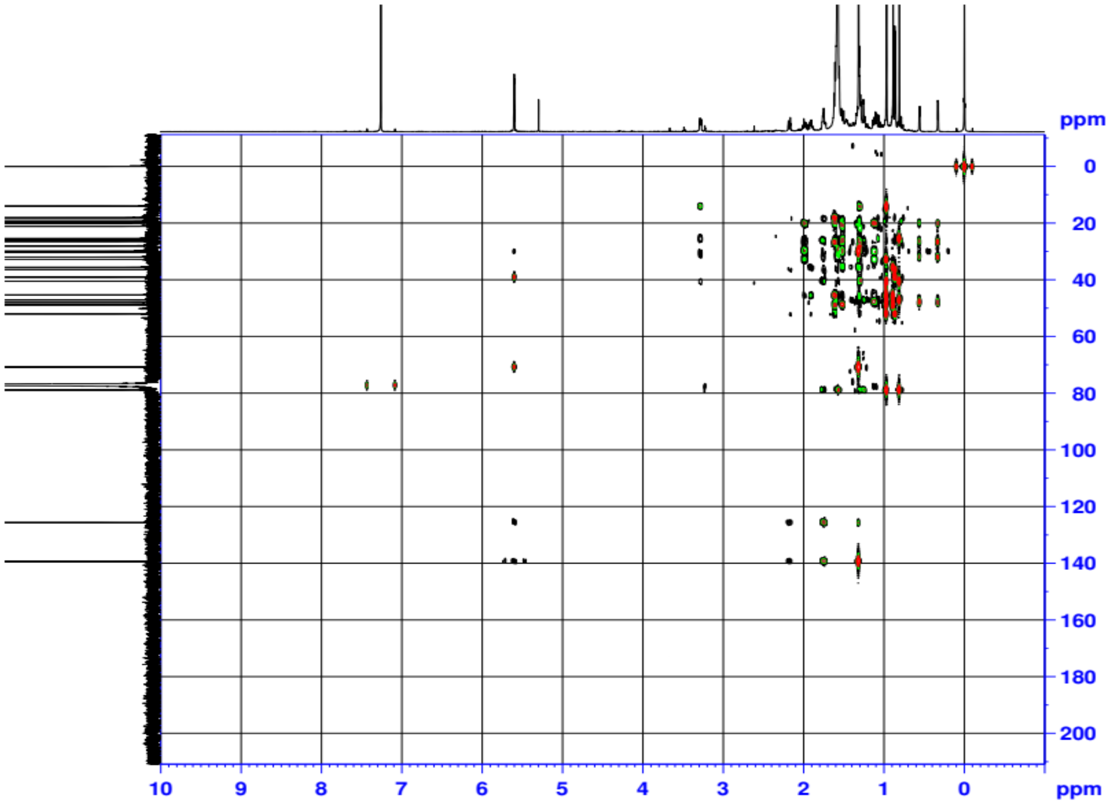

Figure S9. HMBC spectrum ($CDCl_3$) of compound 2

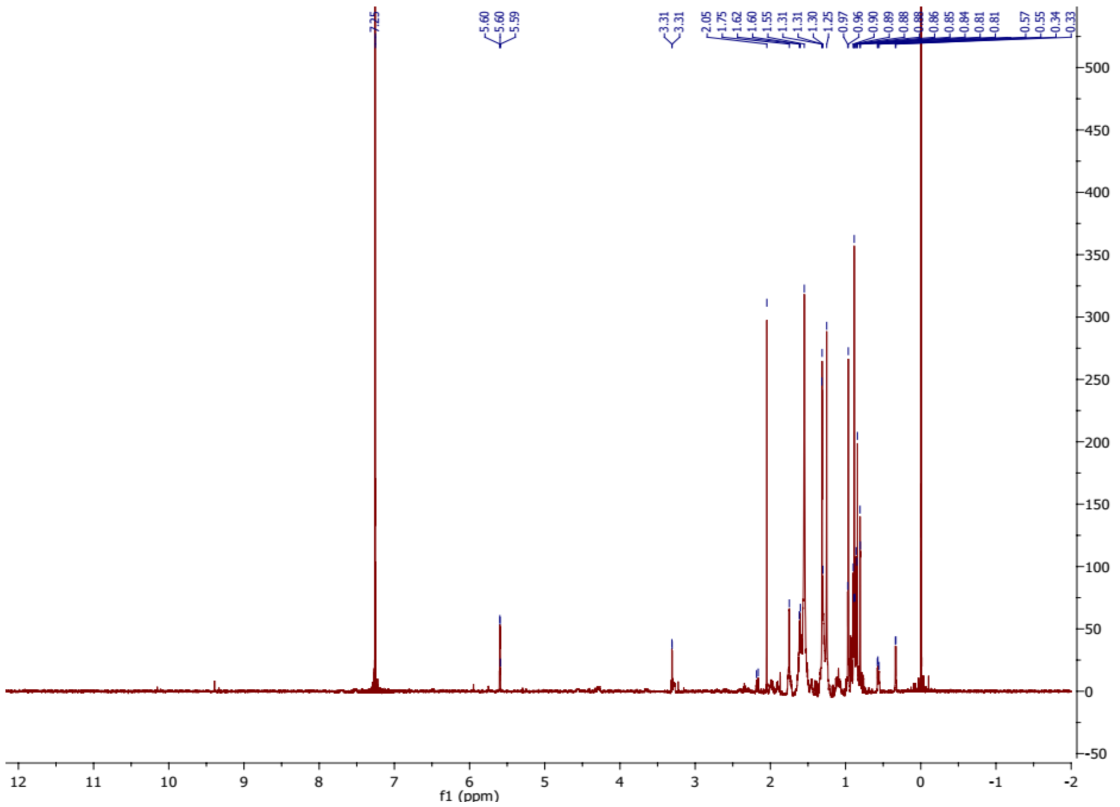

Figure S10. ^{1}H NMR spectrum (600 MHz, CDCl3) of compound 3

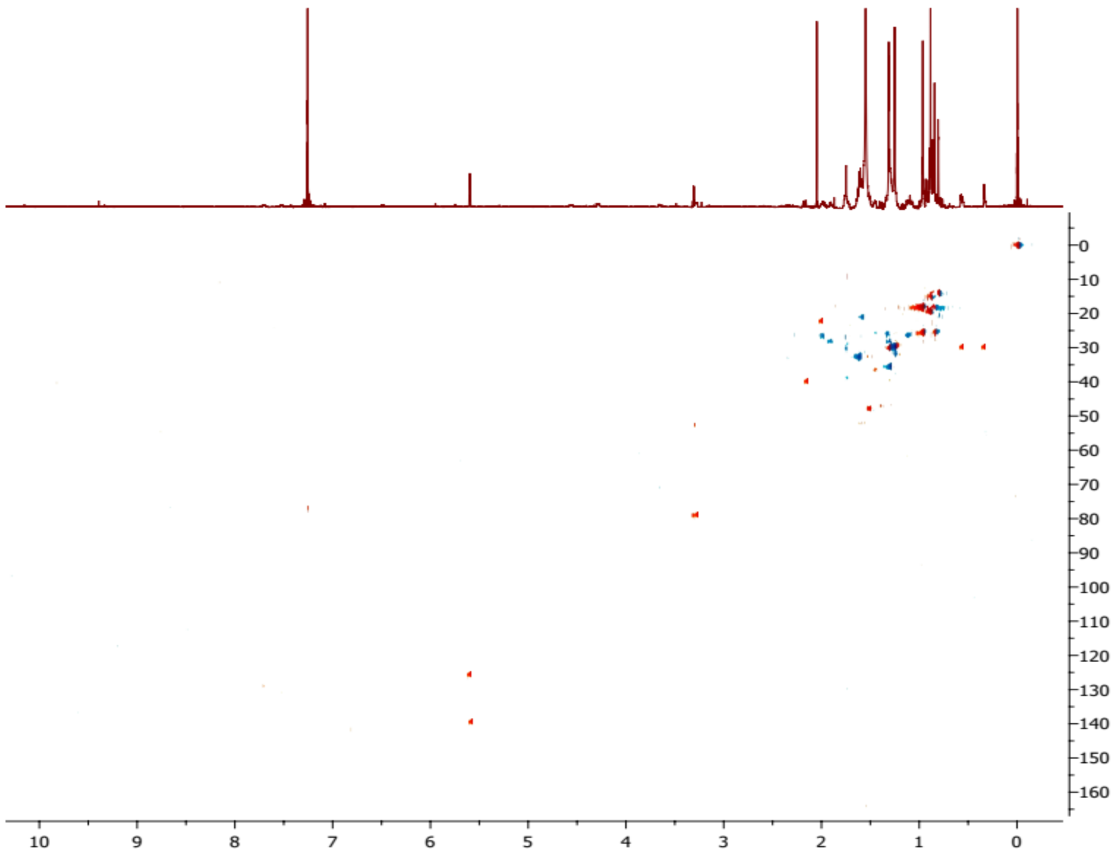

Figure S11. HSQC spectrum (CDCl3) of compound 3

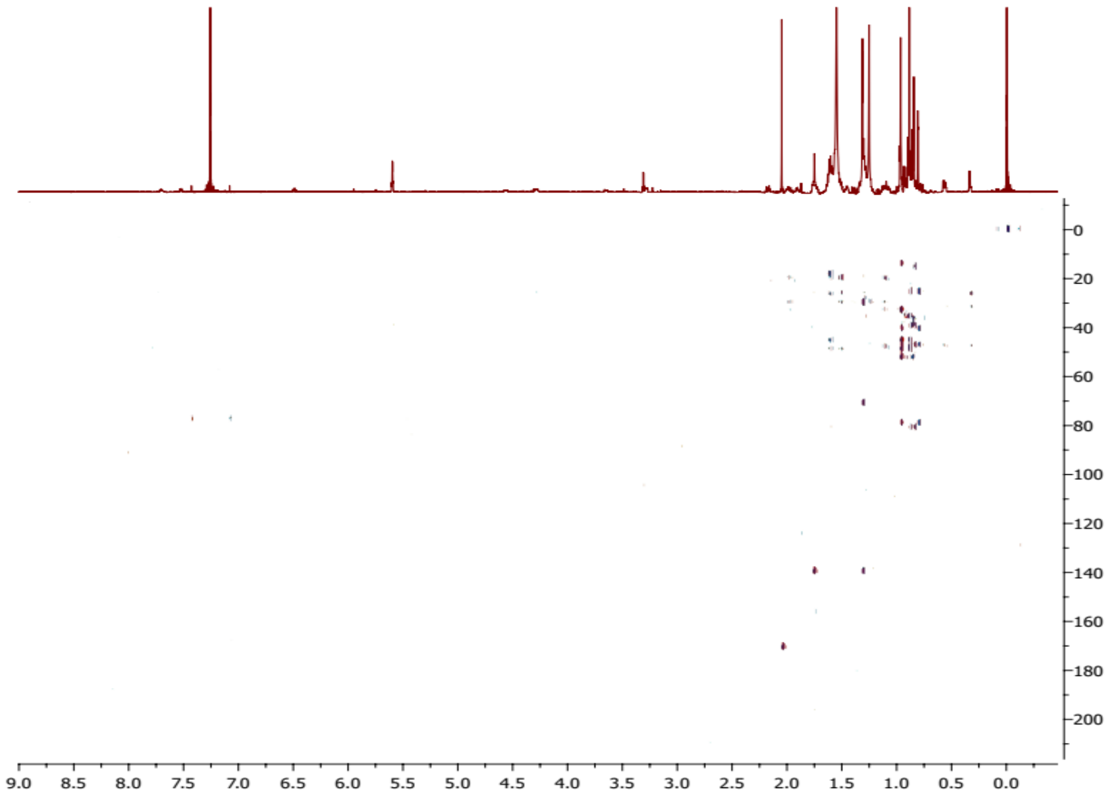

Figure S12. HMBC spectrum ($CDCl_3$) of compound 3

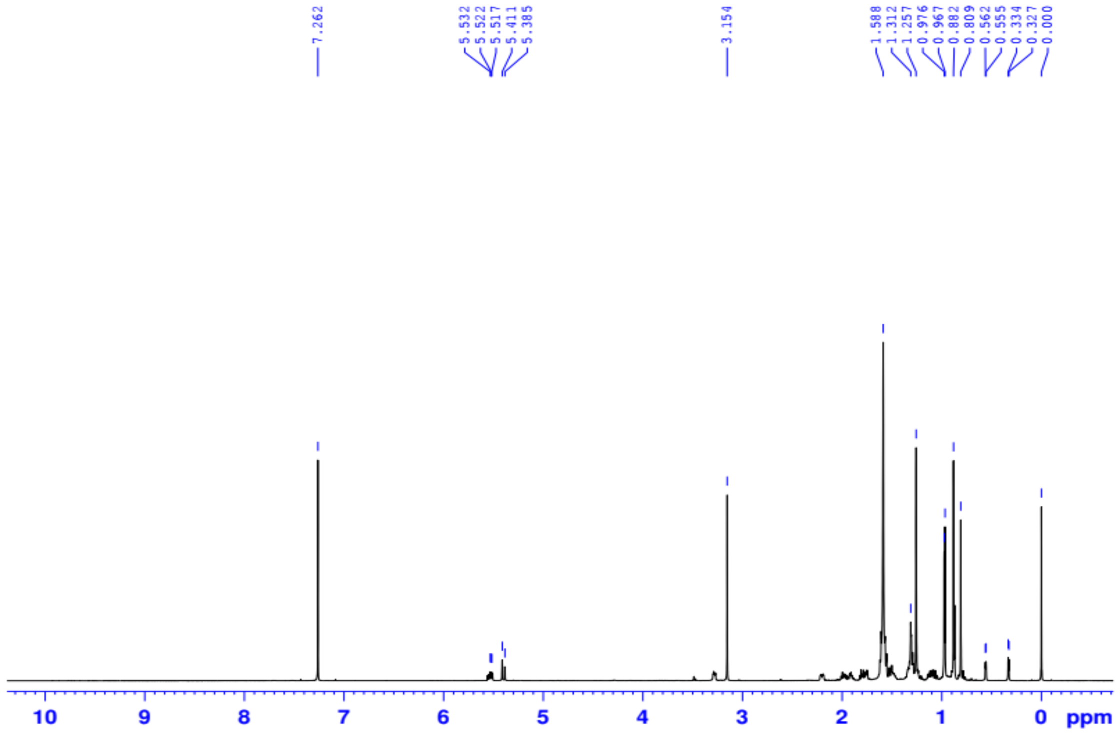

Figure S13. ^{1}H NMR spectrum (600 MHz, $CDCl_3$) of compound 4

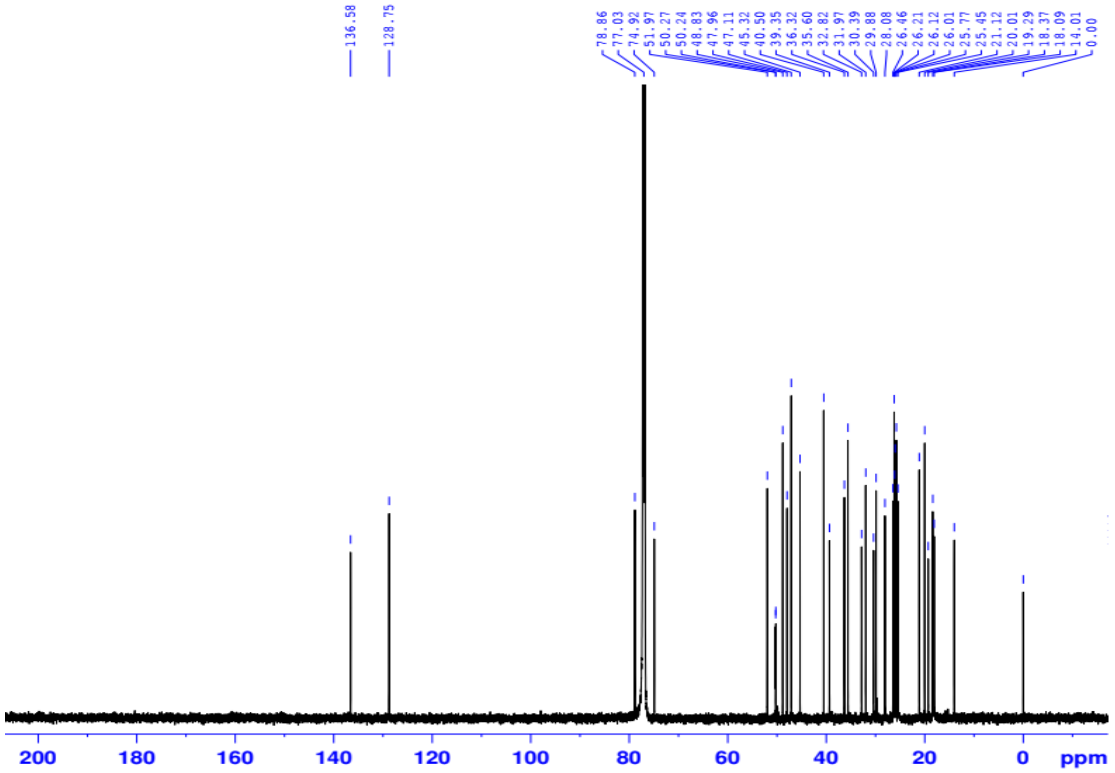

Figure S14. [13]**C NMR spectrum (150 MHz, CDCl**$_3$**) of compound 4**

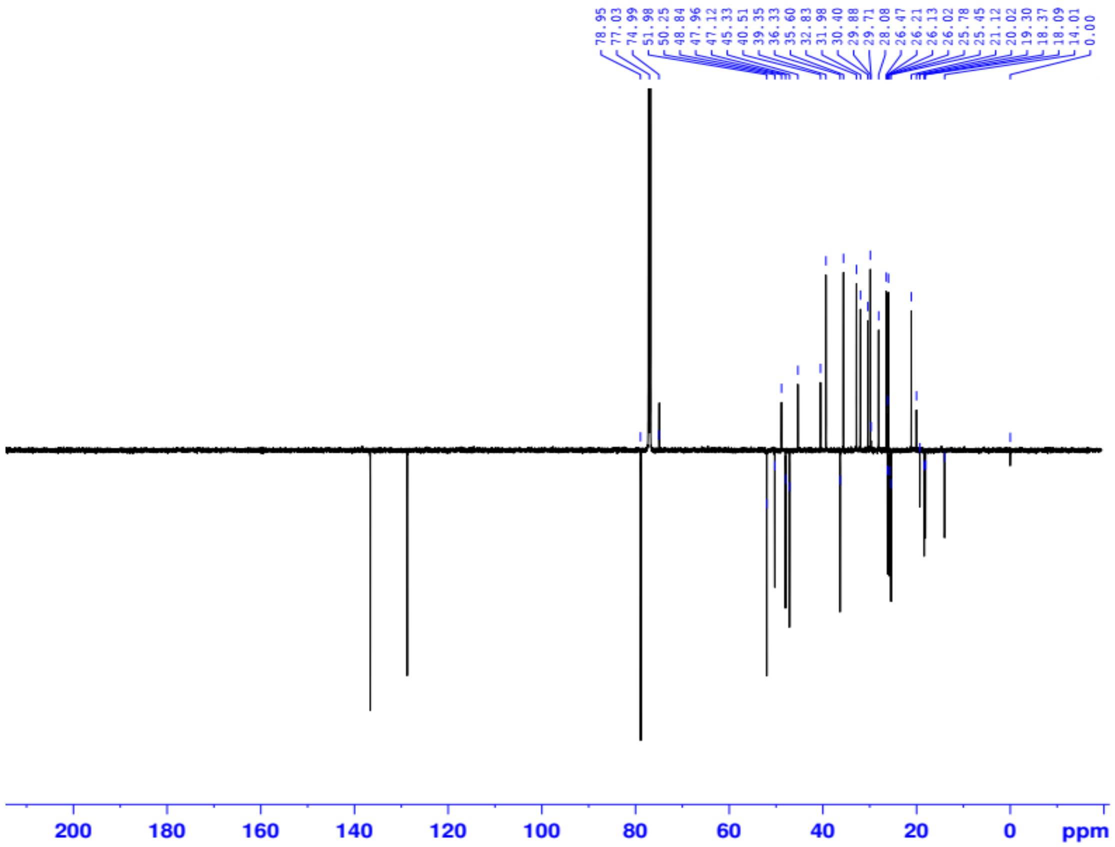

Figure S15. APT spectrum (150 MHz, CDCl$_3$**) of compound 4**

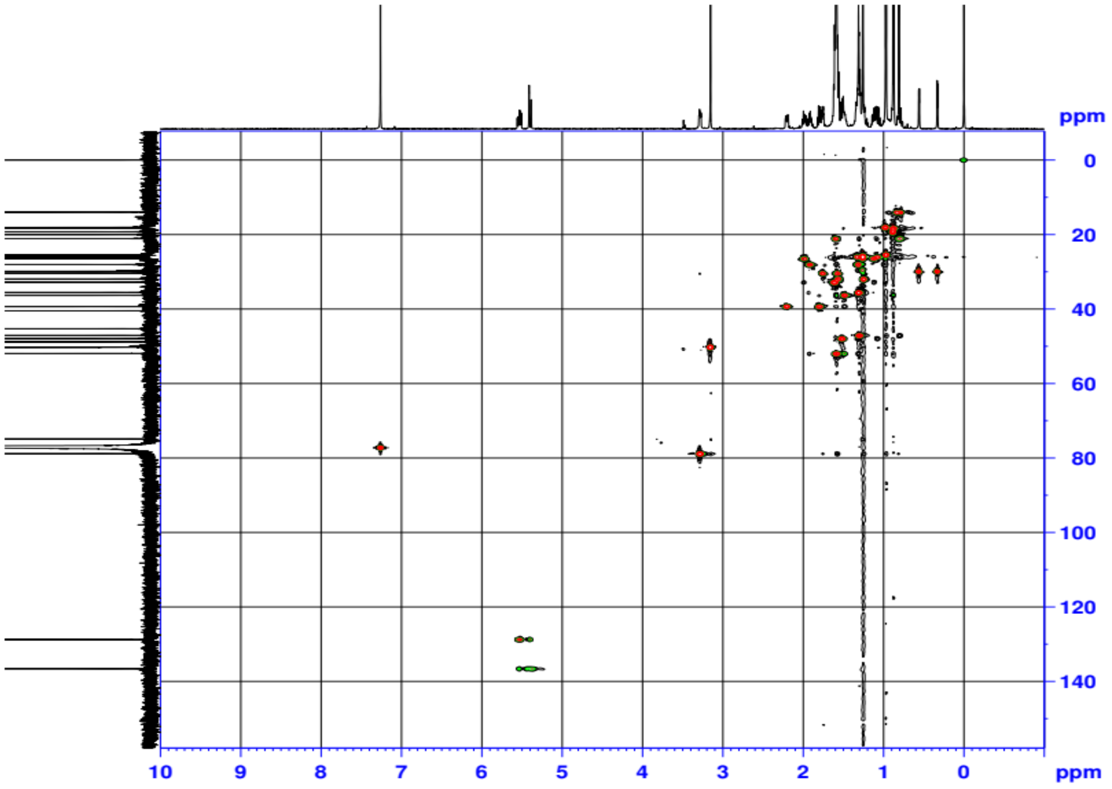

Figure S16. HSQC spectrum ($CDCl_3$) of compound 4

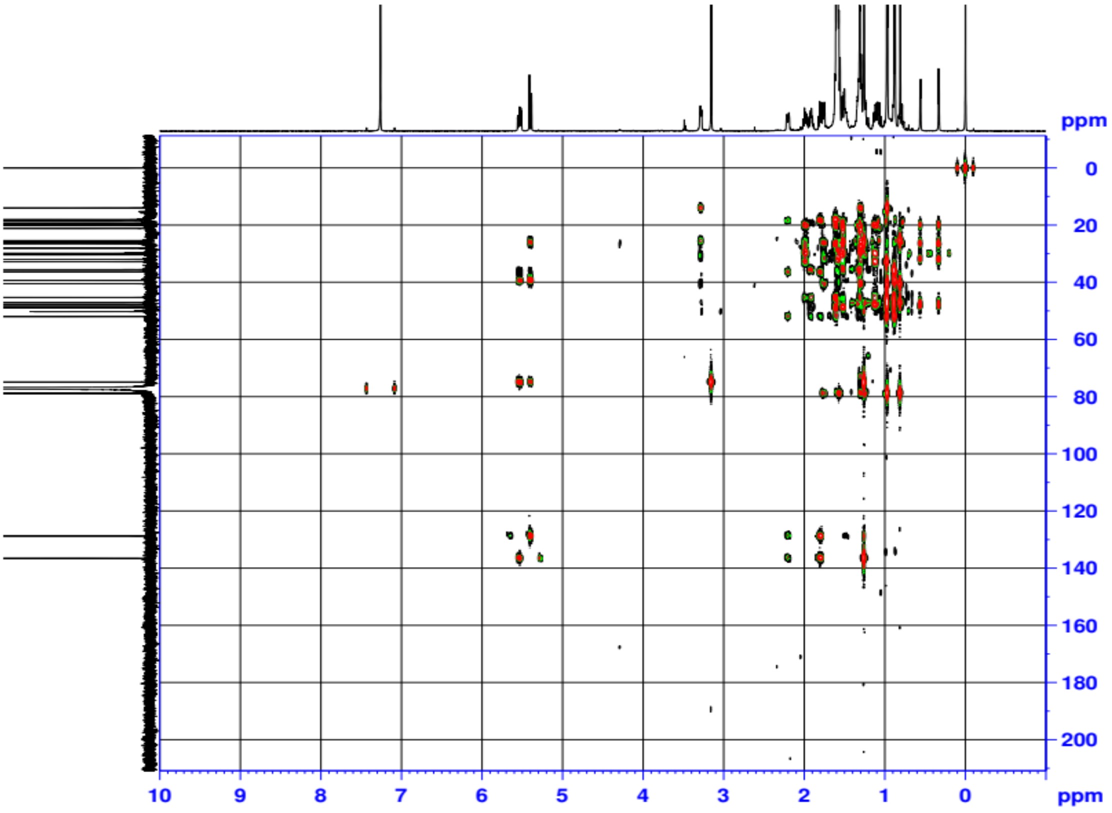

Figure S17. HMBC spectrum ($CDCl_3$) of compound 4

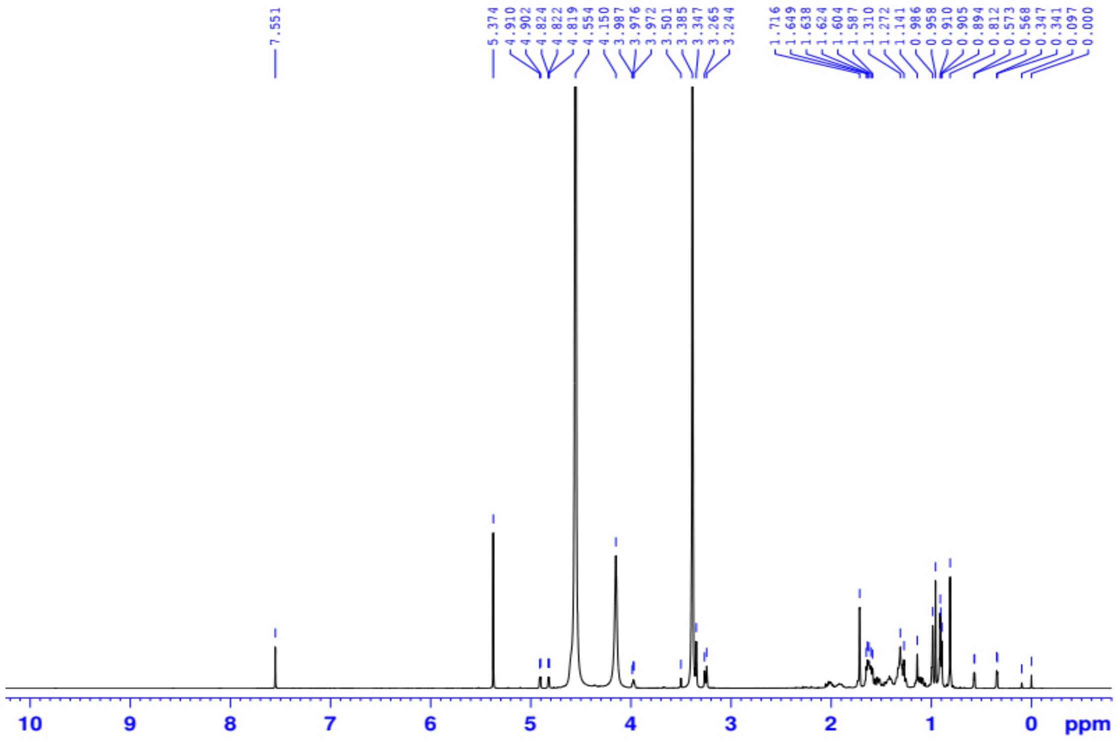

Figure S18. **^{1}H NMR spectrum (600 MHz, CD_3OD & $CDCl_3$) of compound 5**

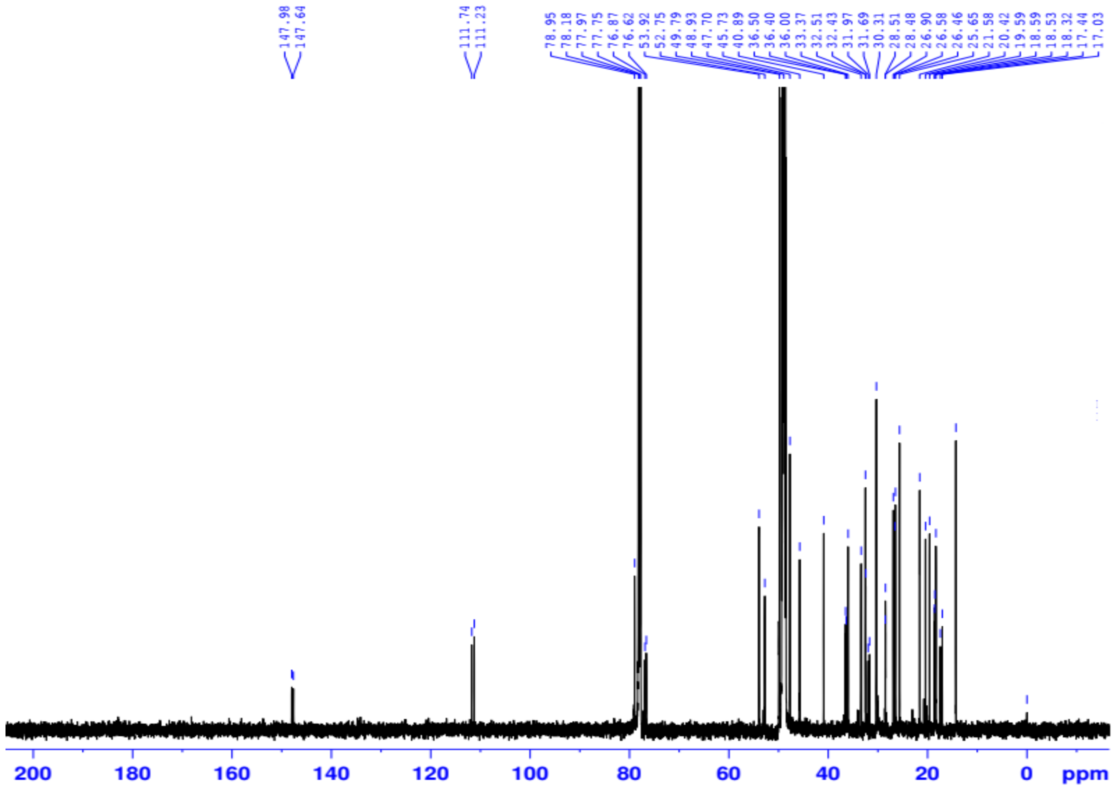

Figure S19. **^{13}C NMR spectrum (150 MHz, CD_3OD & $CDCl_3$) of compound 5**

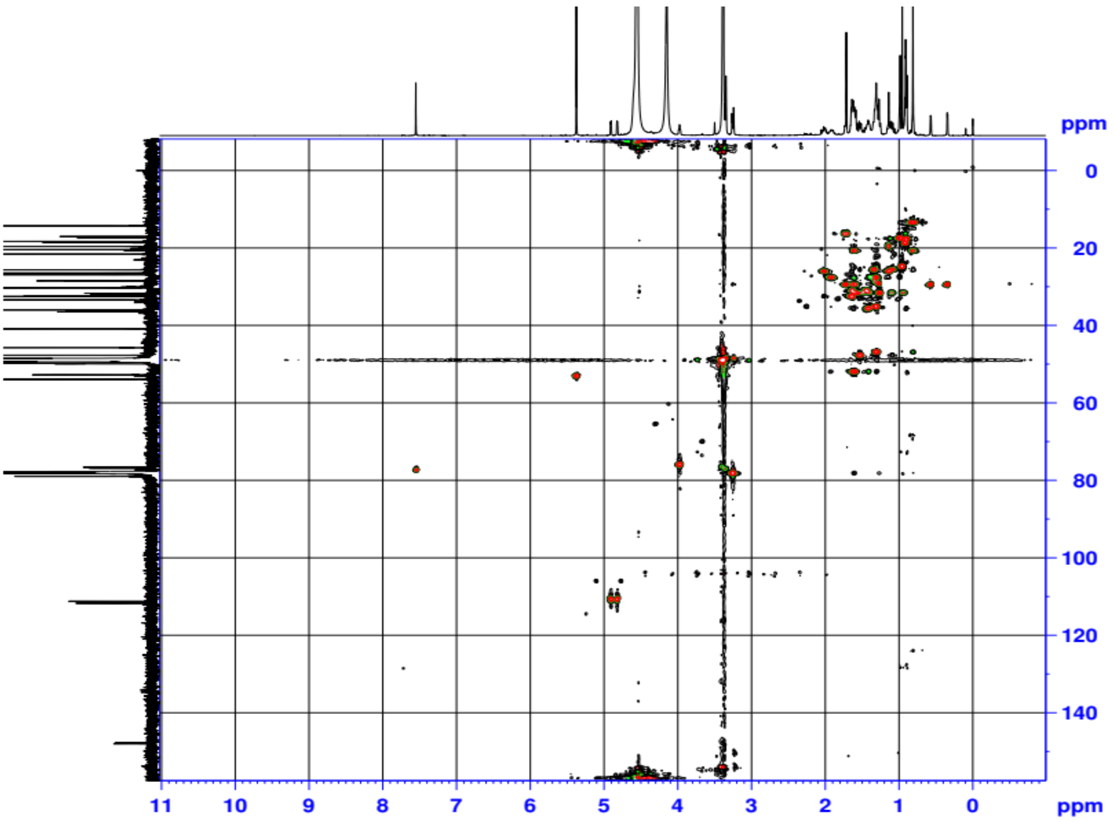

Figure S20. HSQC spectrum (CD_3OD & $CDCl_3$) of compound 5

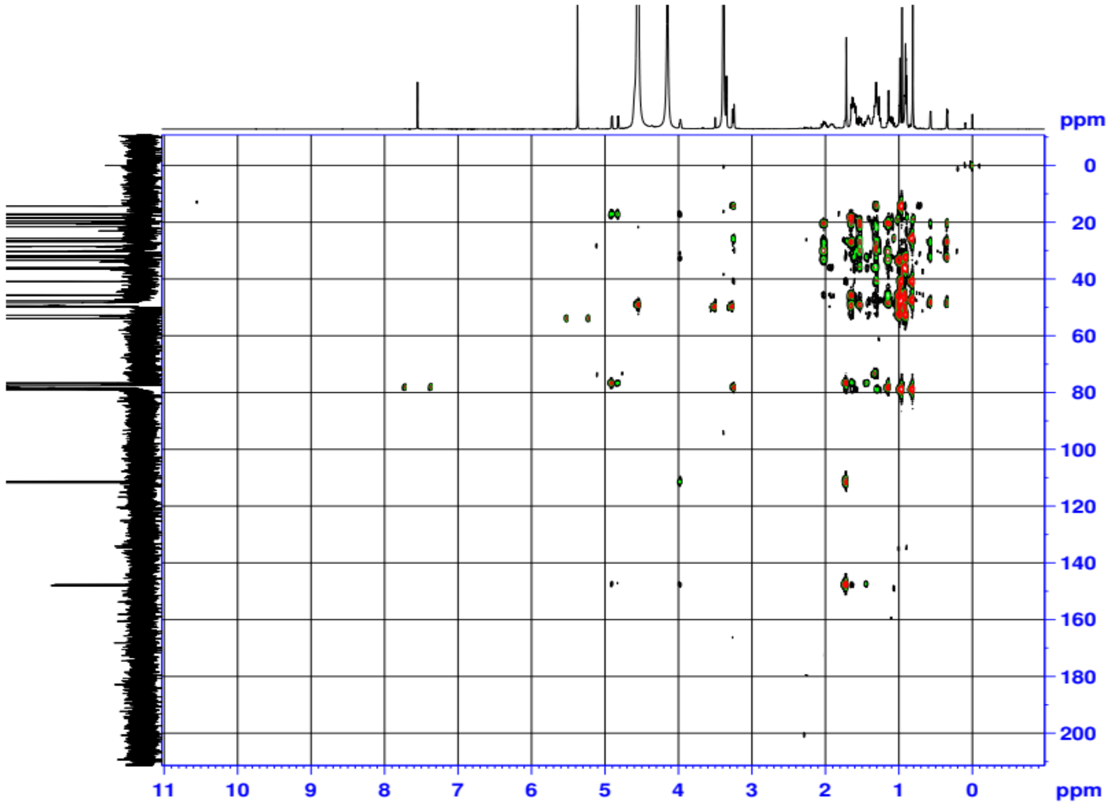

Figure S21. HMBC spectrum (CD_3OD & $CDCl_3$) of compound 5

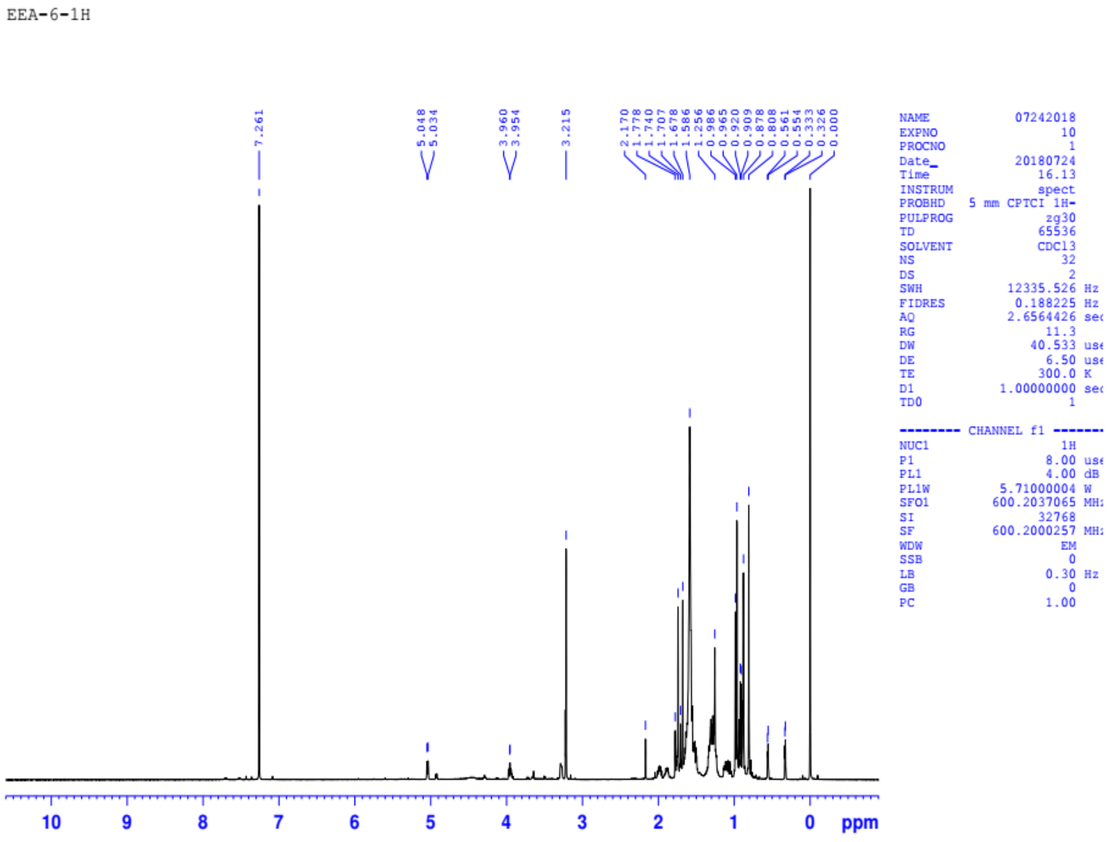

Figure S22. **^{1}H NMR spectrum (600 MHz, $CDCl_3$) of compound 6**

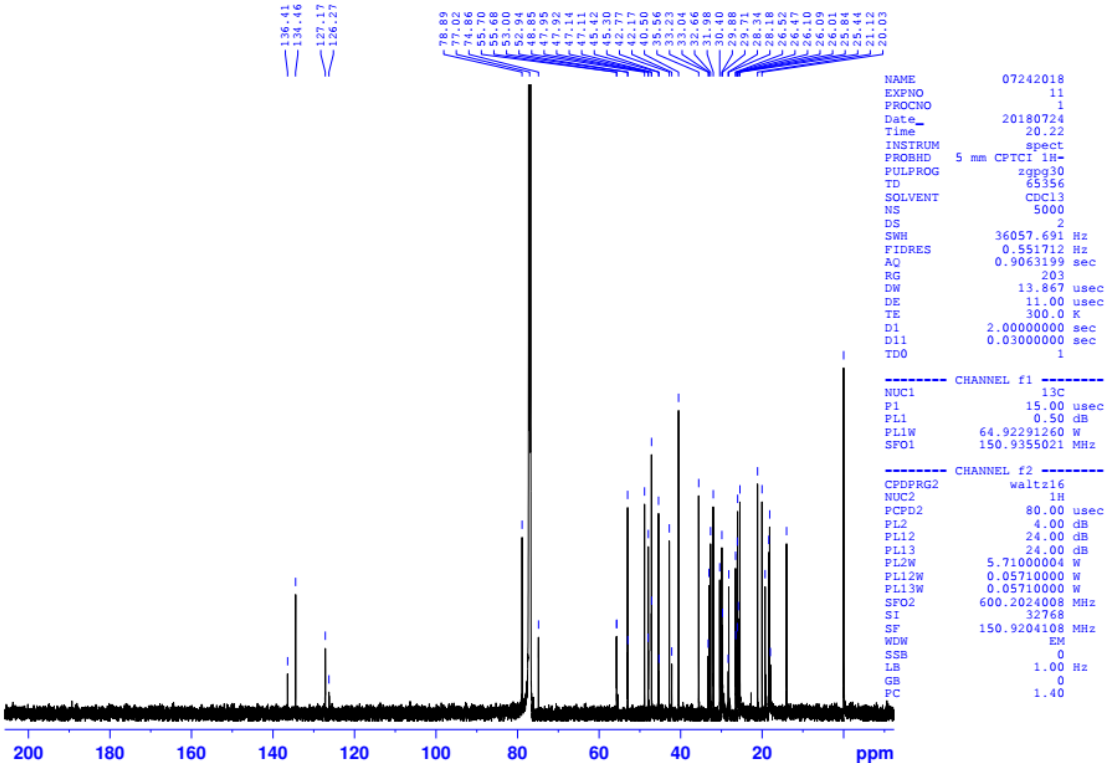

Figure S23. **^{13}C NMR spectrum (150 MHz, $CDCl_3$) of compound 6**

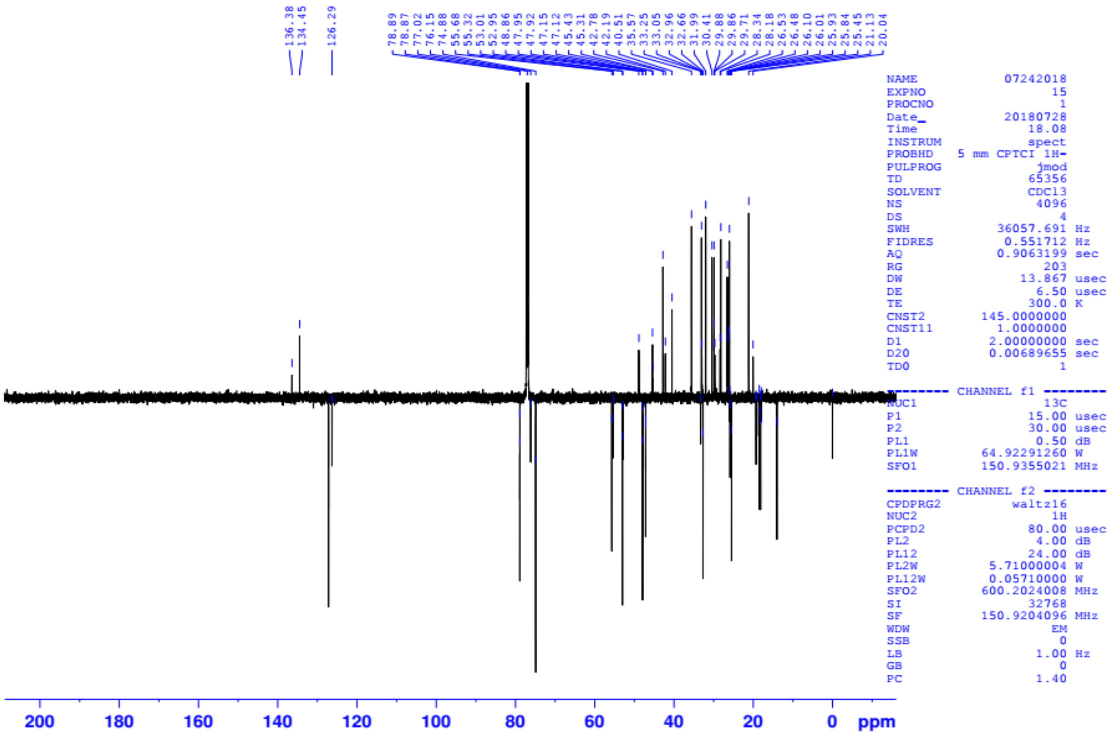

Figure S24. APT spectrum (150 MHz, CDCl$_3$) of compound 6

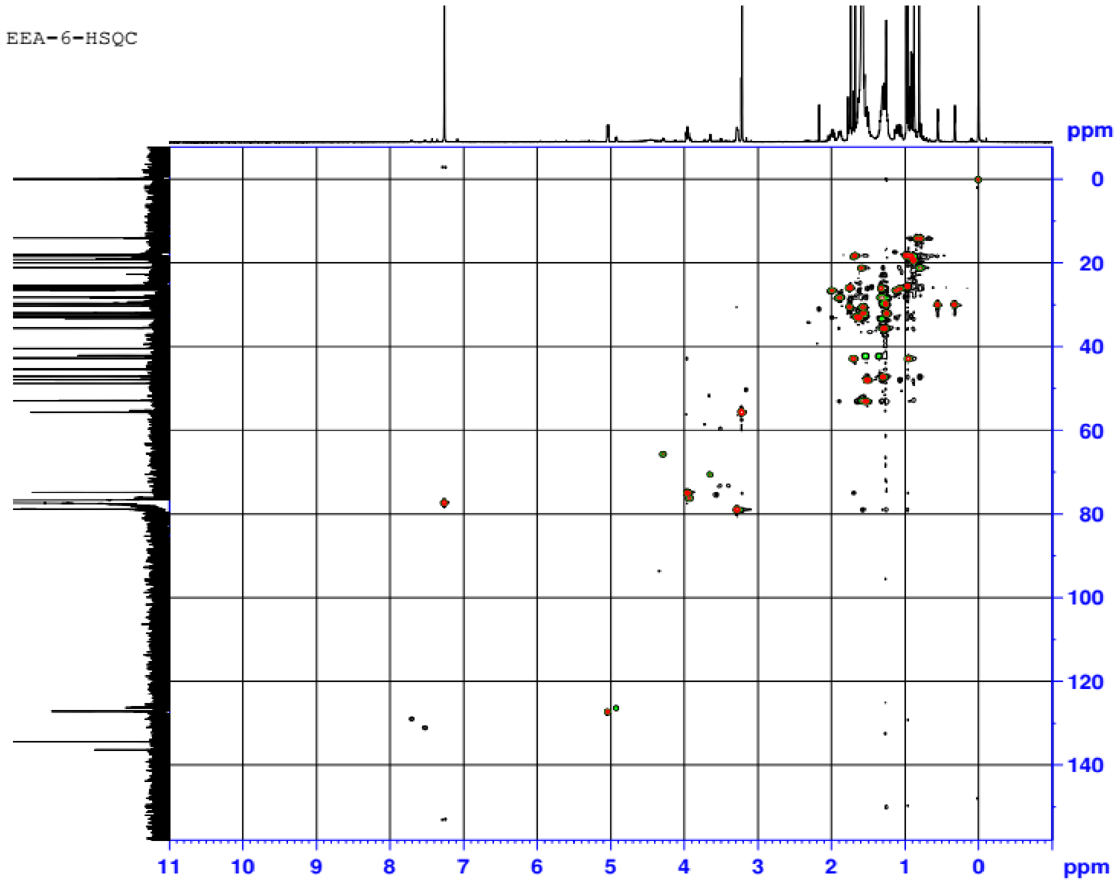

Figure S25. HSQC spectrum (CDCl$_3$) of compound 6

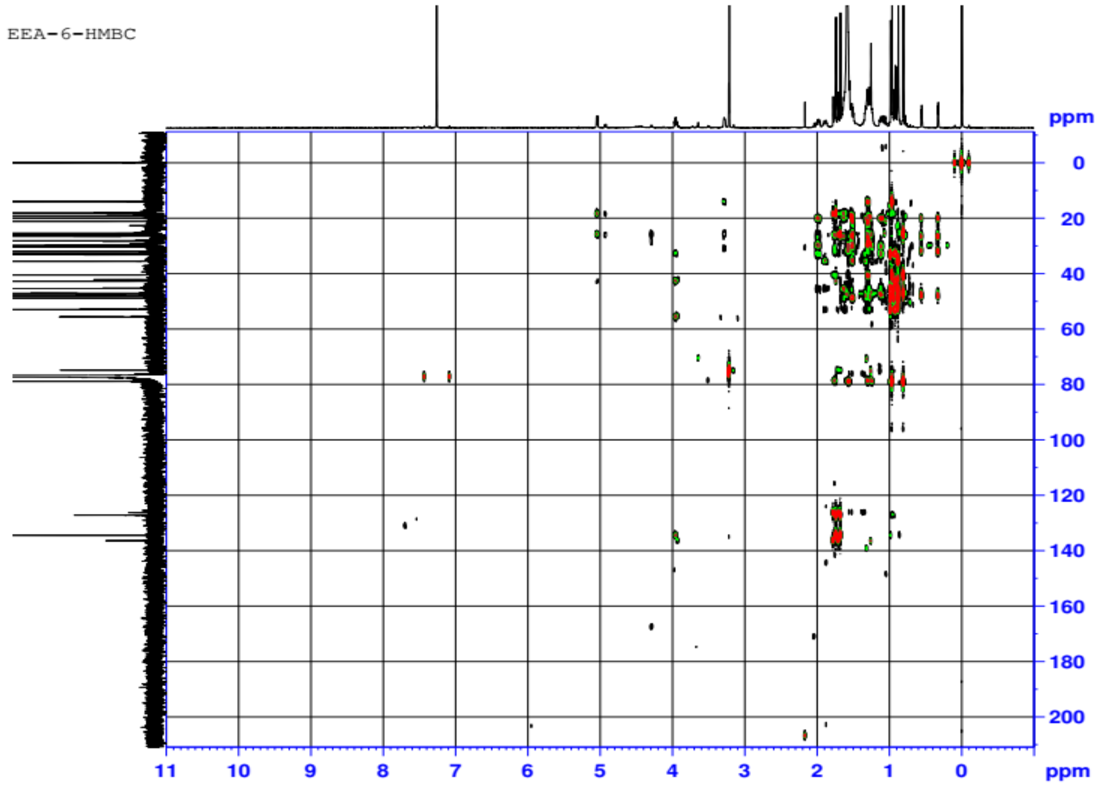

Figure S26. HMBC spectrum (CDCl3) of compound 6

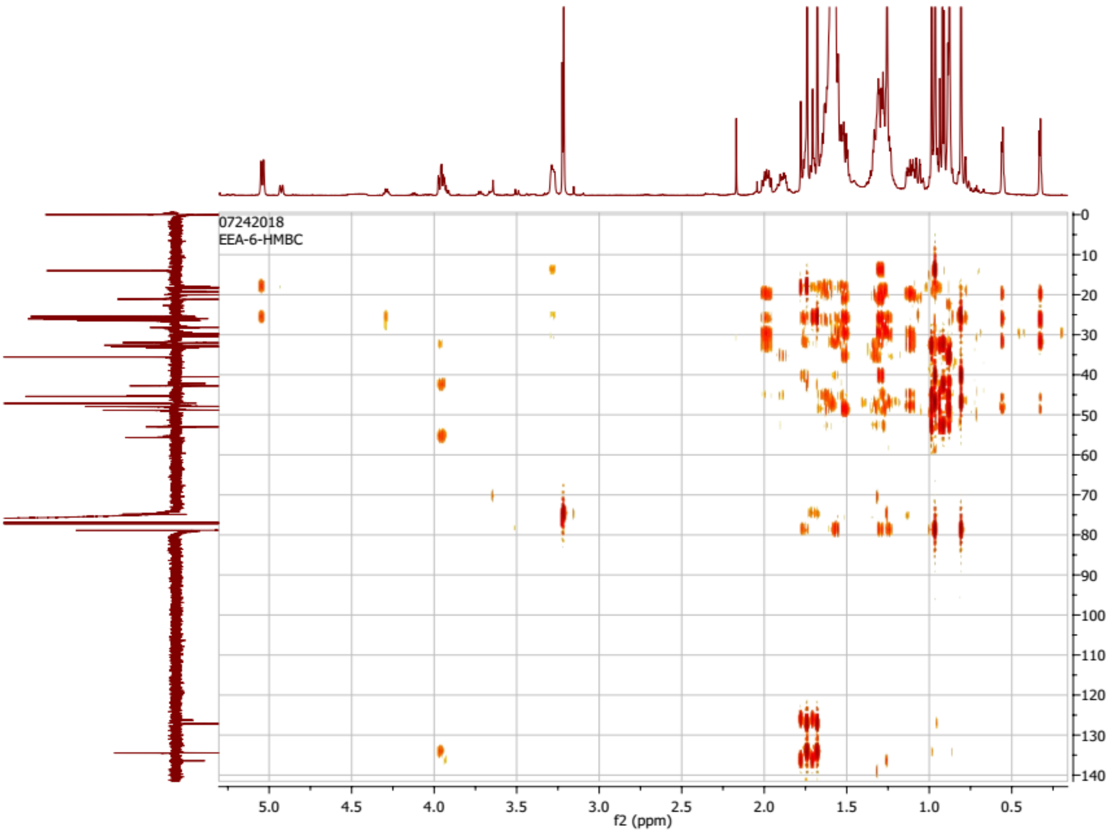

Figure S26-1. Expanded HMBC spectrum (CDCl3) of compound 6

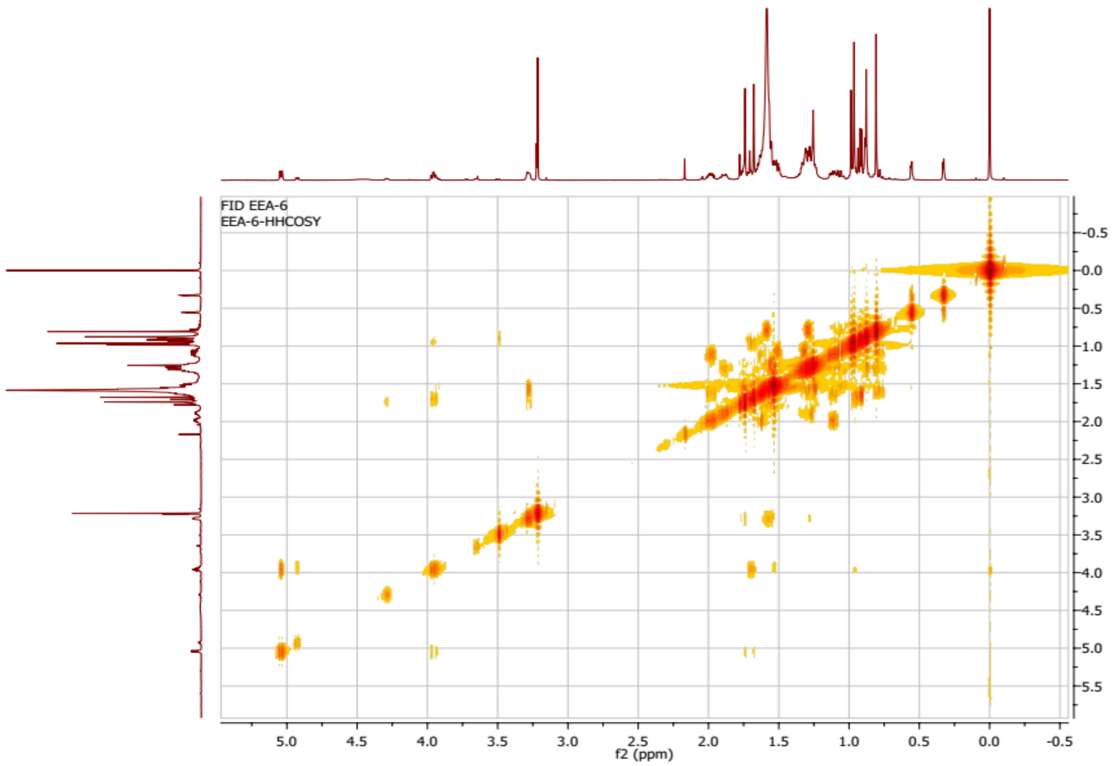

Figure S27. ^1H-^1H COSY spectrum ($CDCl_3$) of compound 6

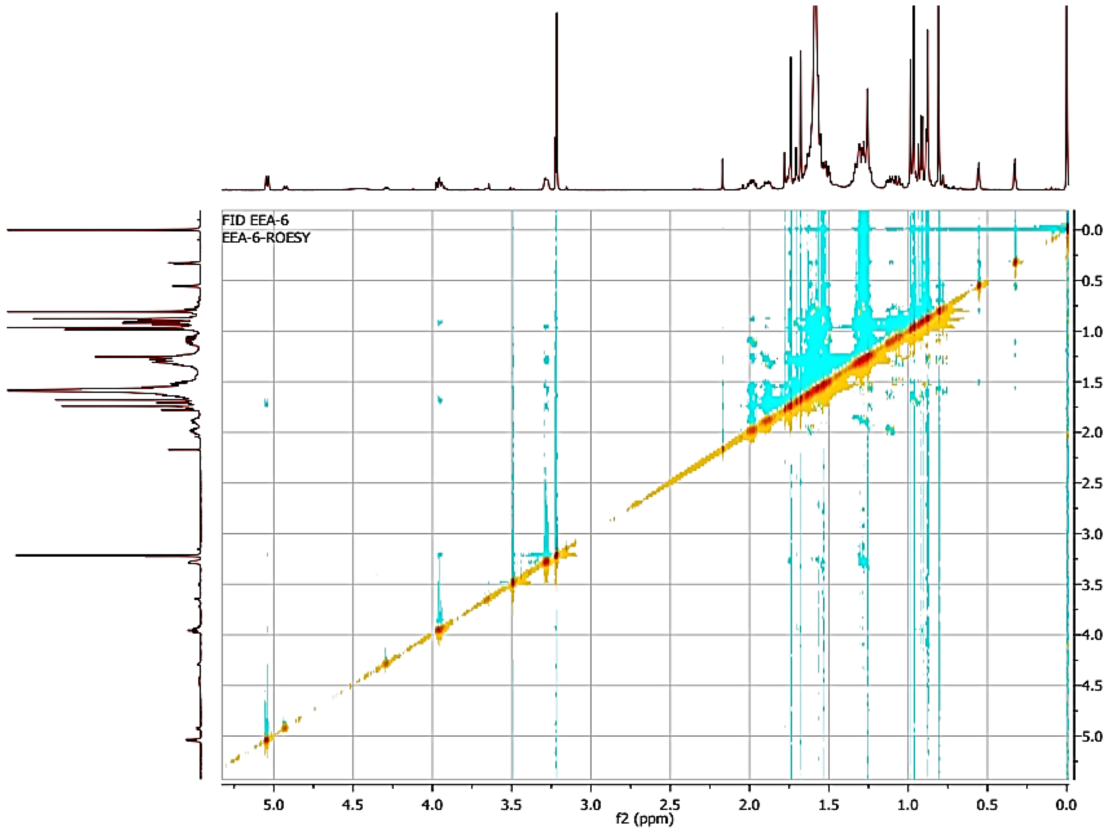

Figure S28. ROESY spectrum ($CDCl_3$) of compound 6

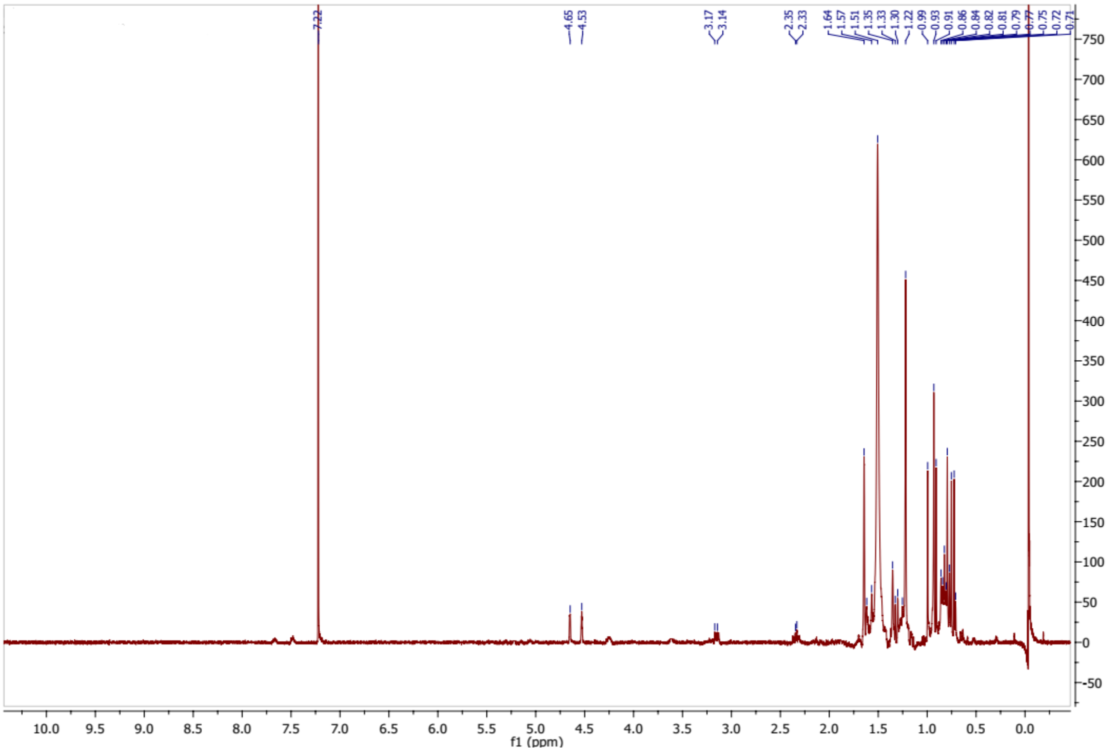

Figure S29. ¹H NMR spectrum (600 MHz, $CDCl_3$) of compound 7

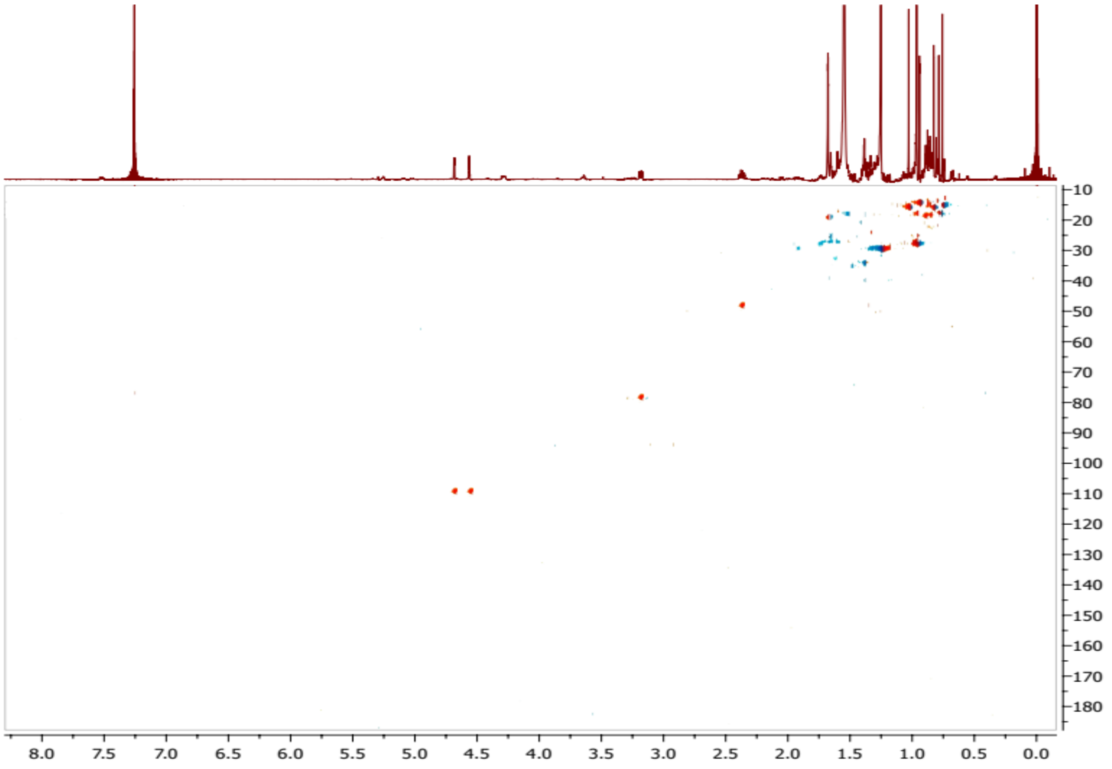

Figure S30. HSQC spectrum ($CDCl_3$) of compound 7

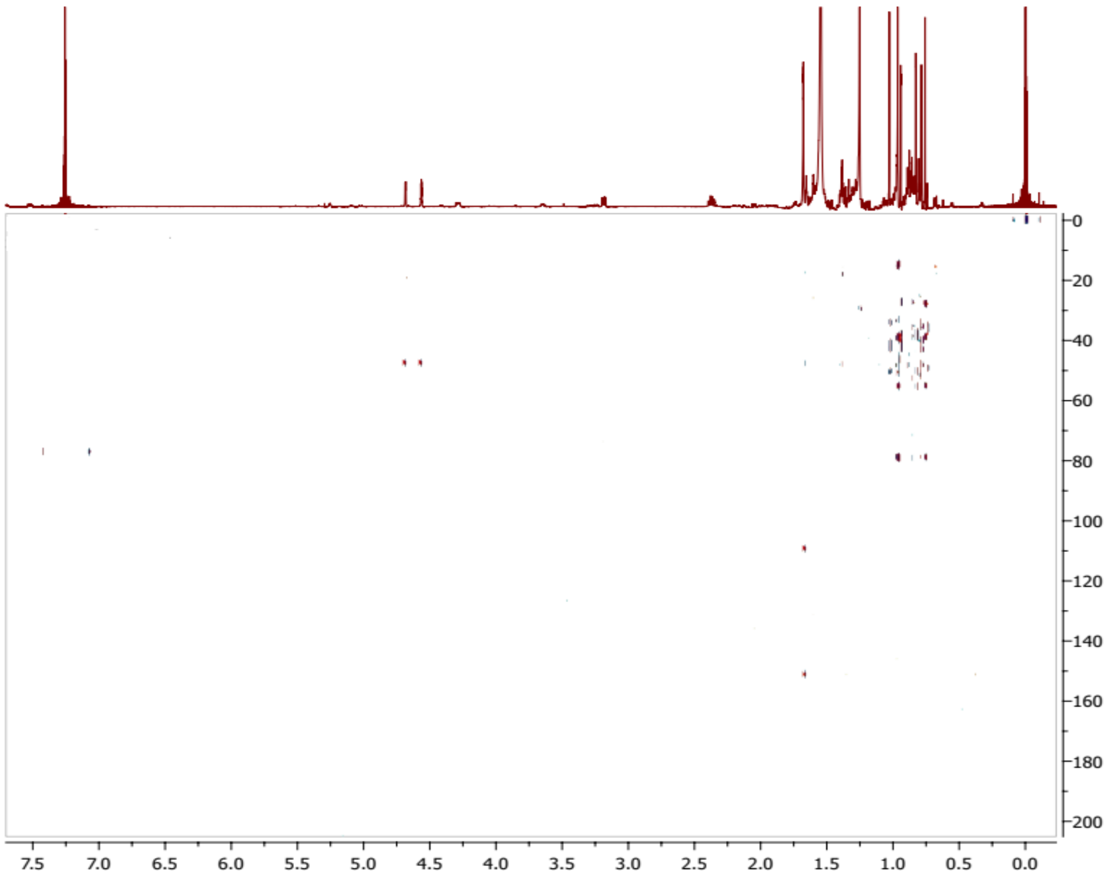

Figure S31. HMBC spectrum ($CDCl_3$) of compound 7

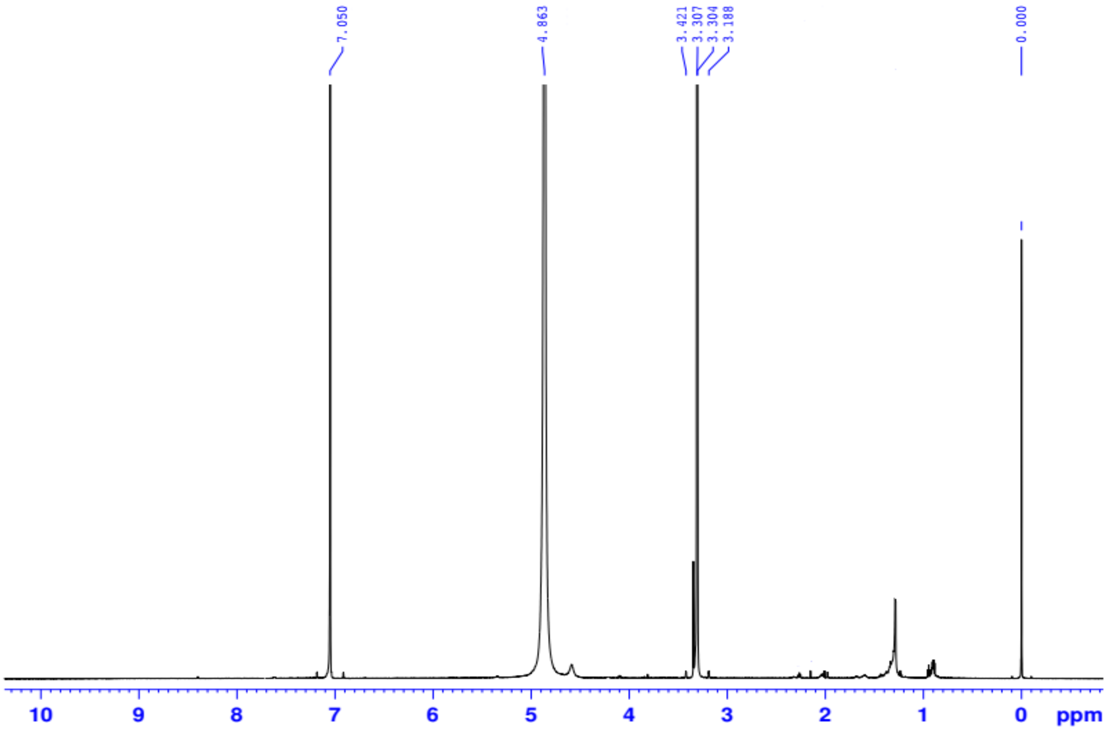

Figure S32. ^1H NMR spectrum (600 MHz, CD_3OD) of compound 8

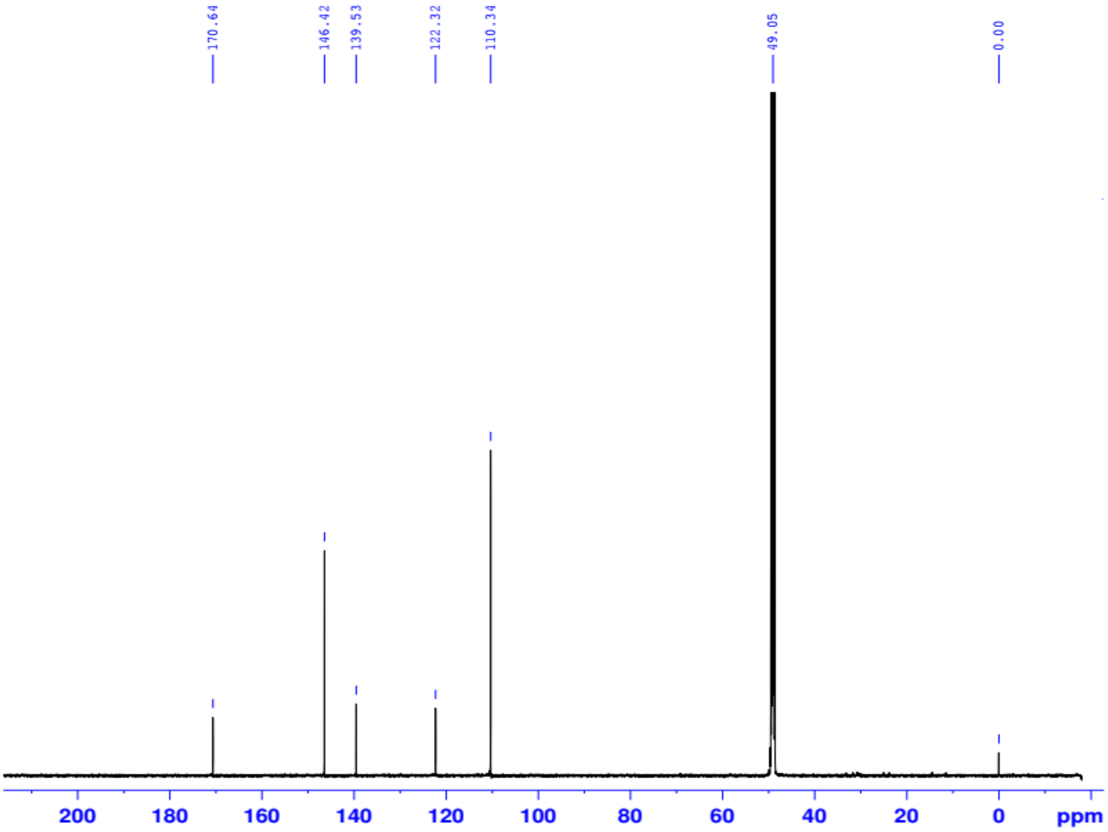

Figure S33. ^{13}C **NMR spectrum (150 MHz,** CD_3OD**) of compound 8**

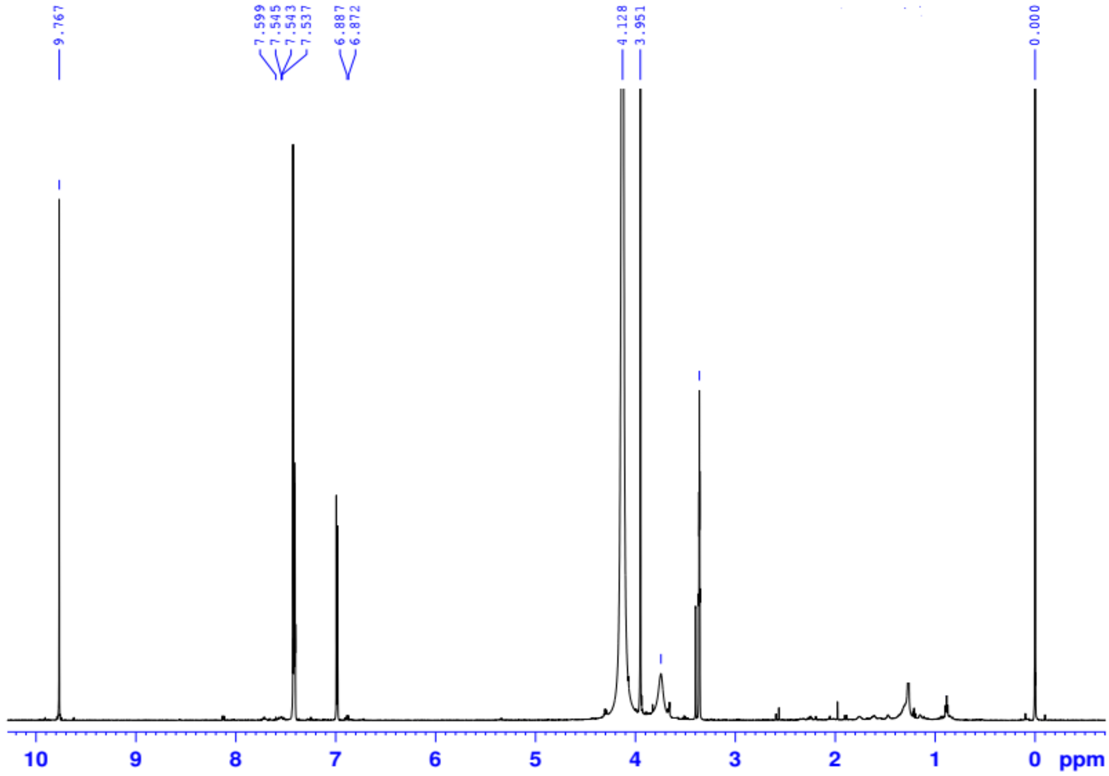

Figure S34. ^{1}H **NMR spectrum (600 MHz,** CD_3OD **&** $CDCl_3$**) of compound 9**

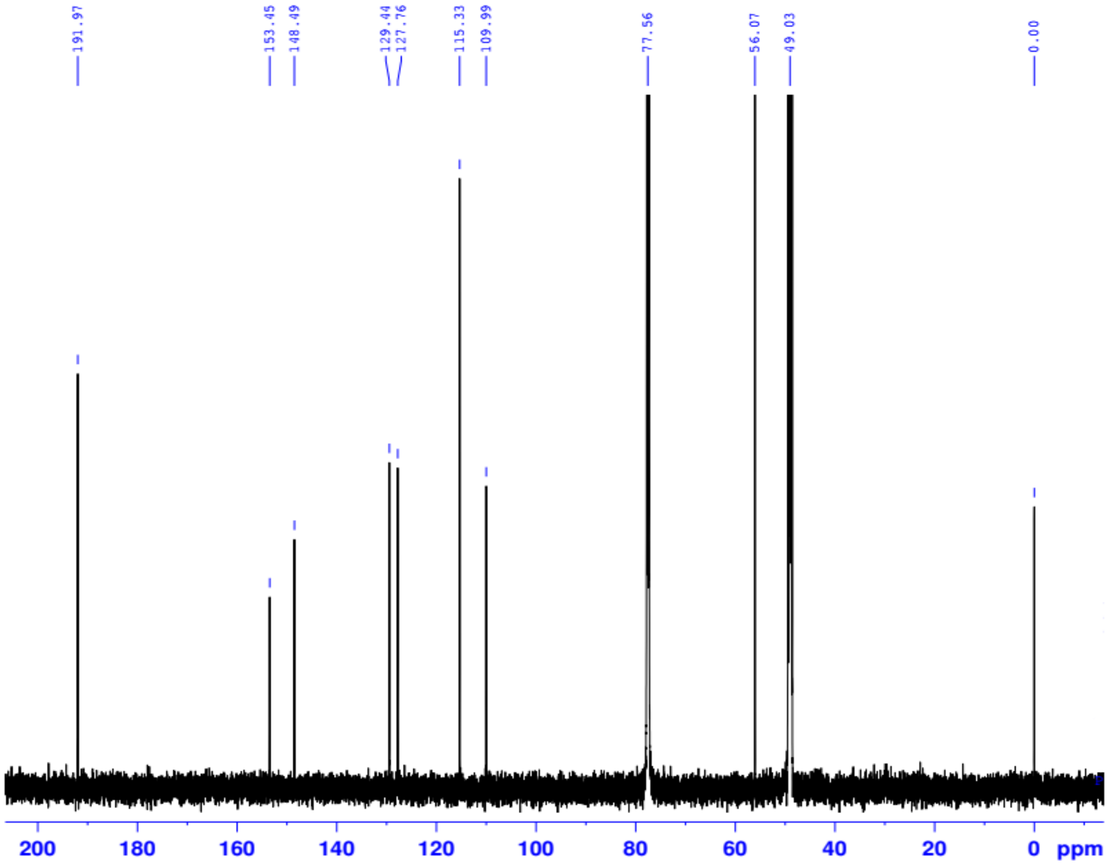

Figure S35. ^{13}C NMR spectrum (150 MHz, CD_3OD & $CDCl_3$) of compound 9

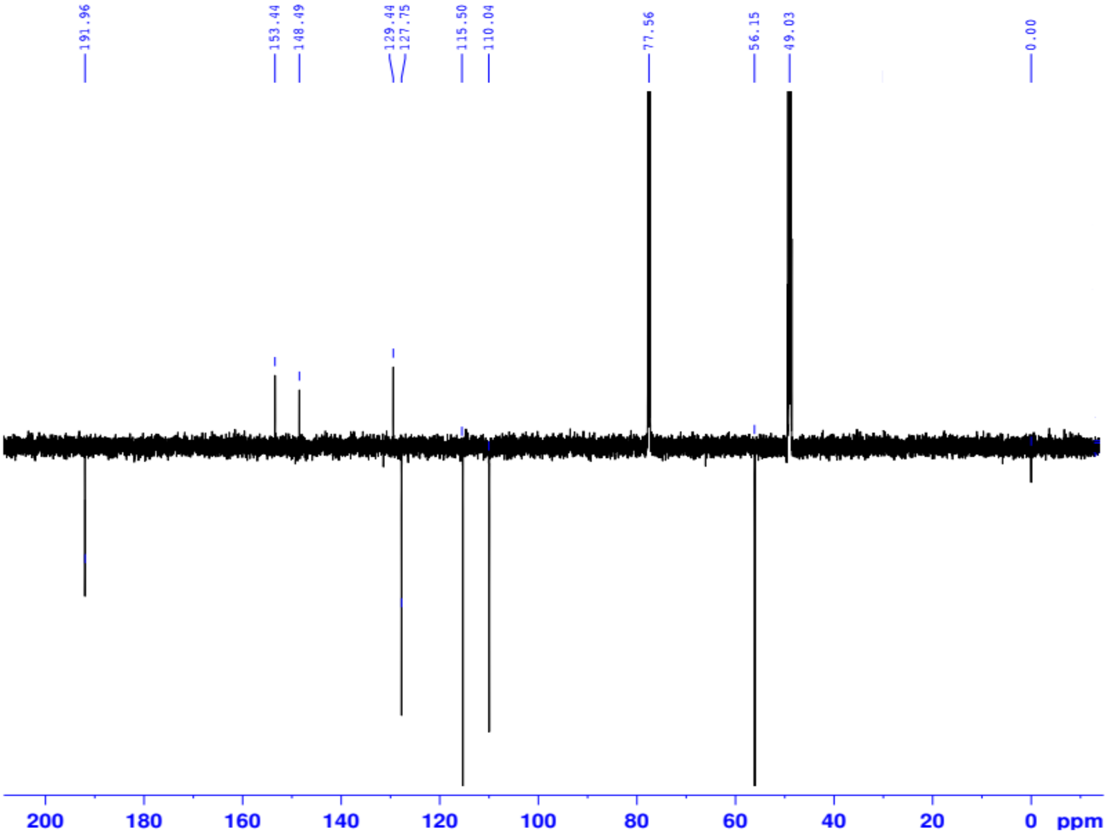

Figure S36. APT spectrum (150 MHz, CD_3OD & $CDCl_3$) of compound 9

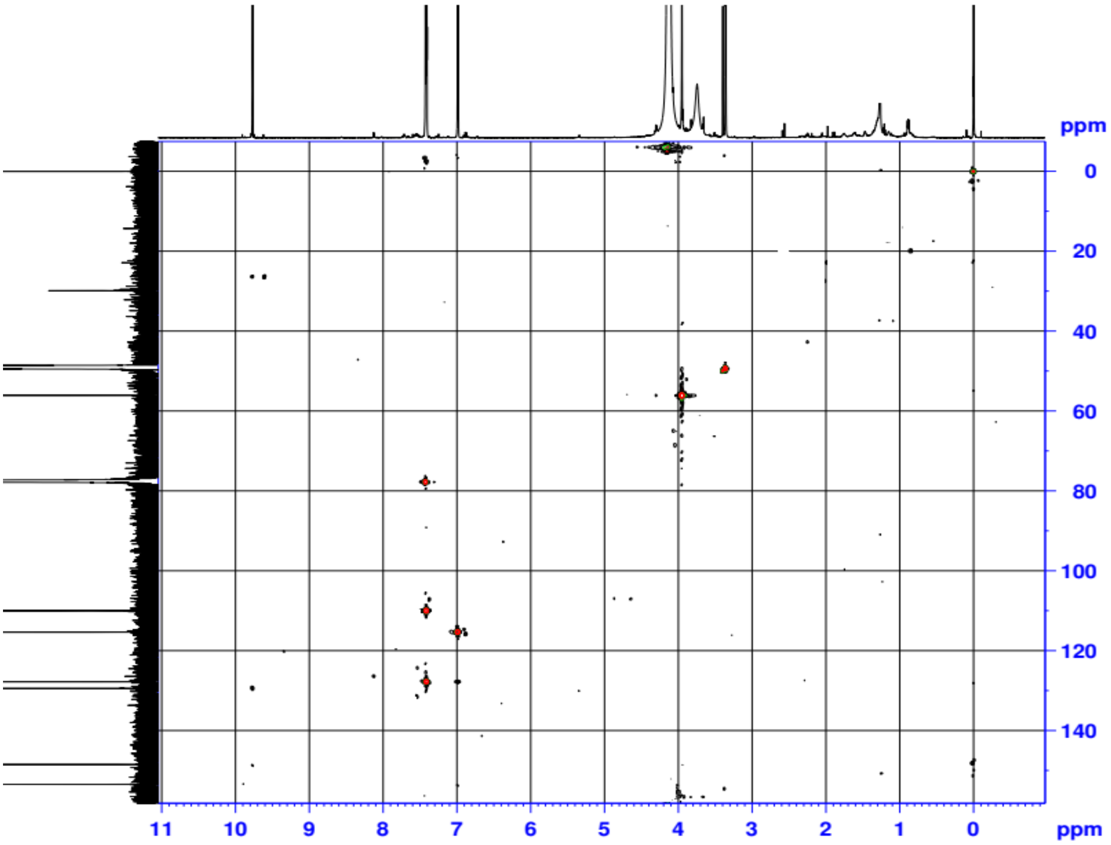

Figure S37. HSQC spectrum (CD_3OD & $CDCl_3$) of compound 9

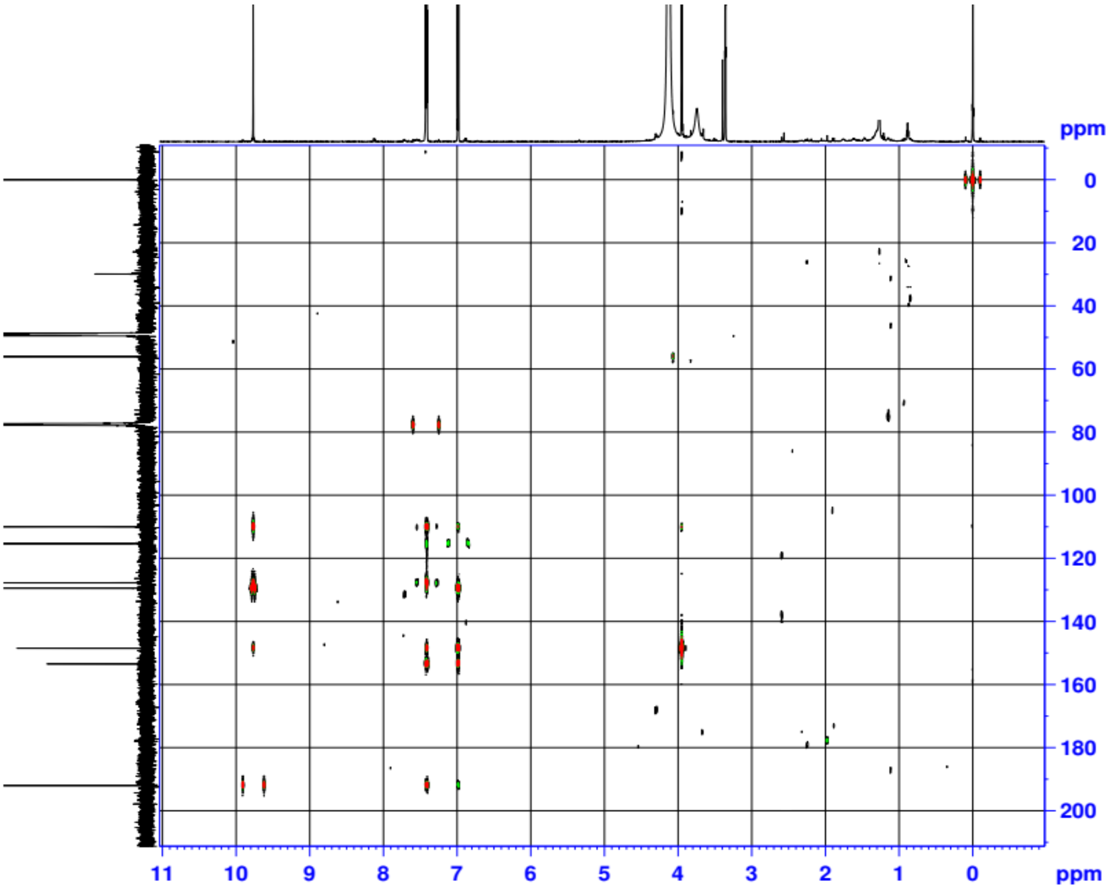

Figure S38. HMBC spectrum (CD_3OD & $CDCl_3$) of compound 9

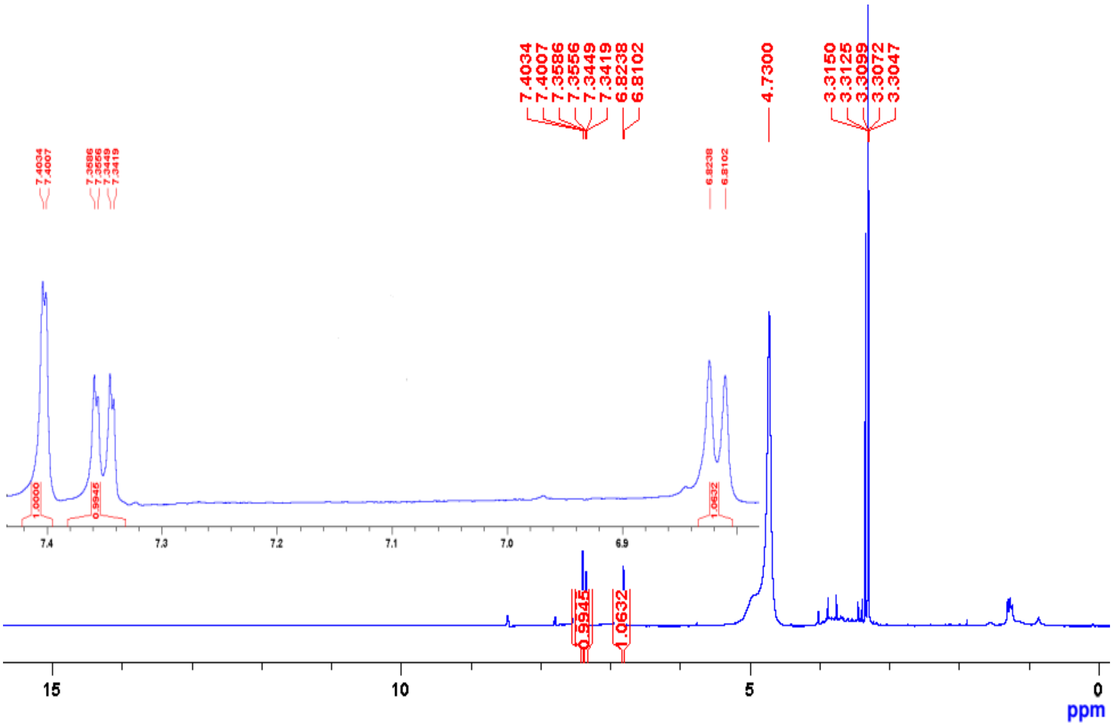

Figure S39. ^1H NMR spectrum (600 MHz, CD_3OD) of compound 10

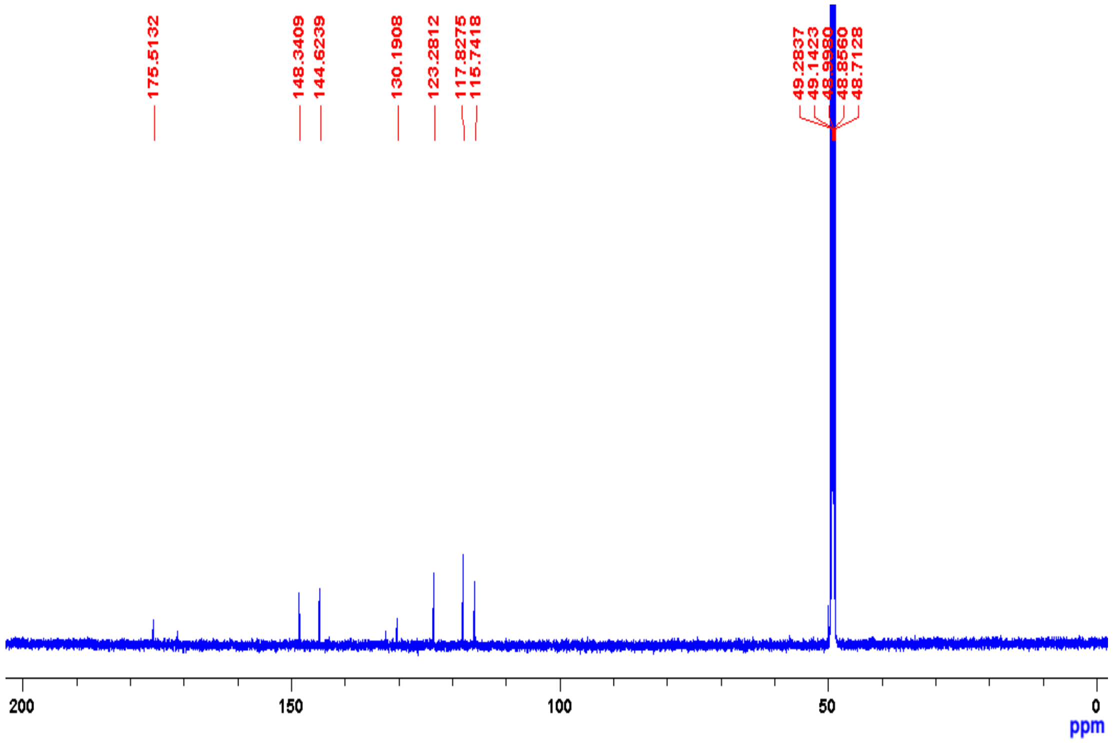

Figure S40. ^{13}C **NMR spectrum (150 MHz, CD$_3$OD) of compound 10**

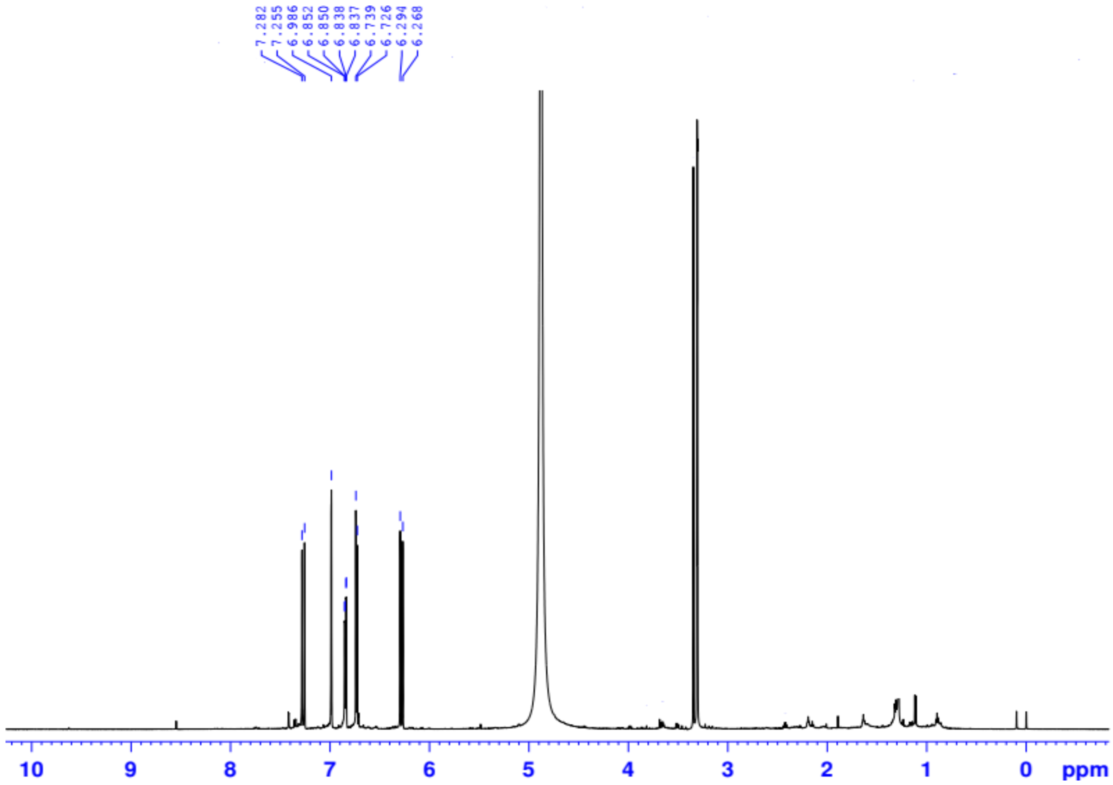

Figure S41. ^{1}H **NMR spectrum (600 MHz, CD$_3$OD) of compound 11**

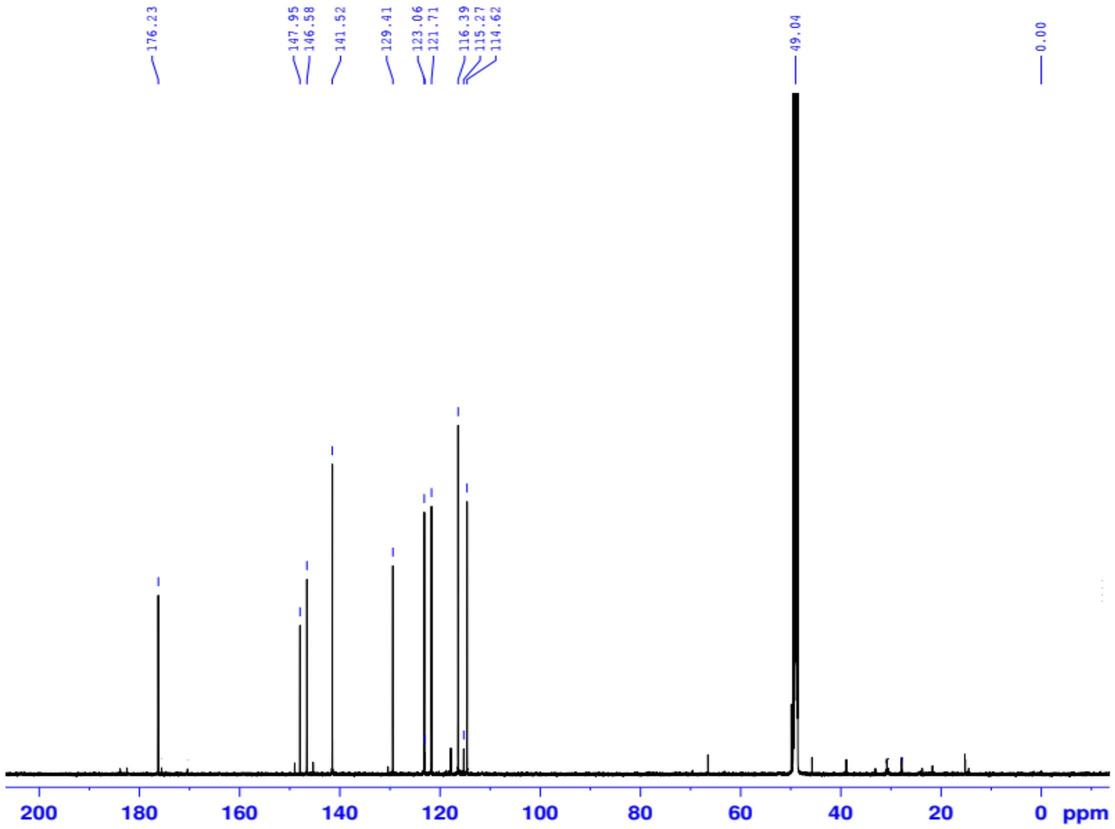

Figure S42. ^{13}C **NMR spectrum (150 MHz, CD_3OD) of compound 11**

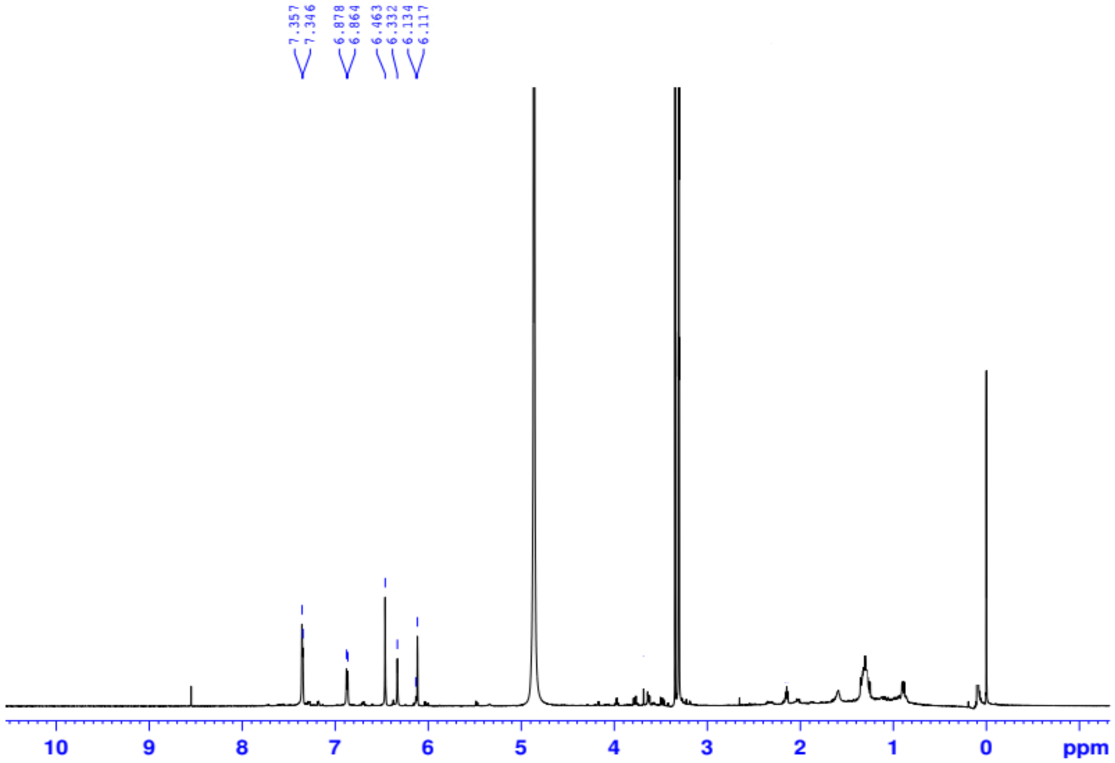

Figure S43. ^{1}H **NMR spectrum (600 MHz, CD_3OD) of compound 12**

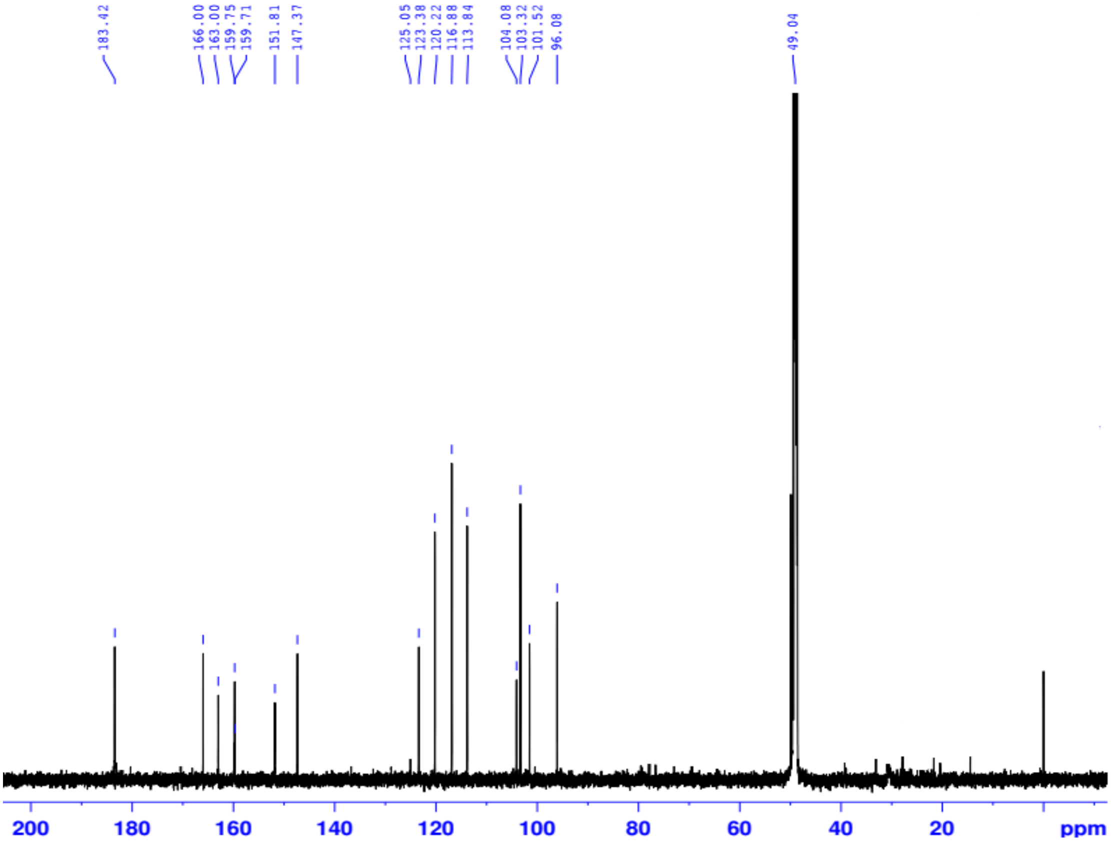

Figure S44. ^{13}C NMR spectrum (150 MHz, CD_3OD) of compound 12

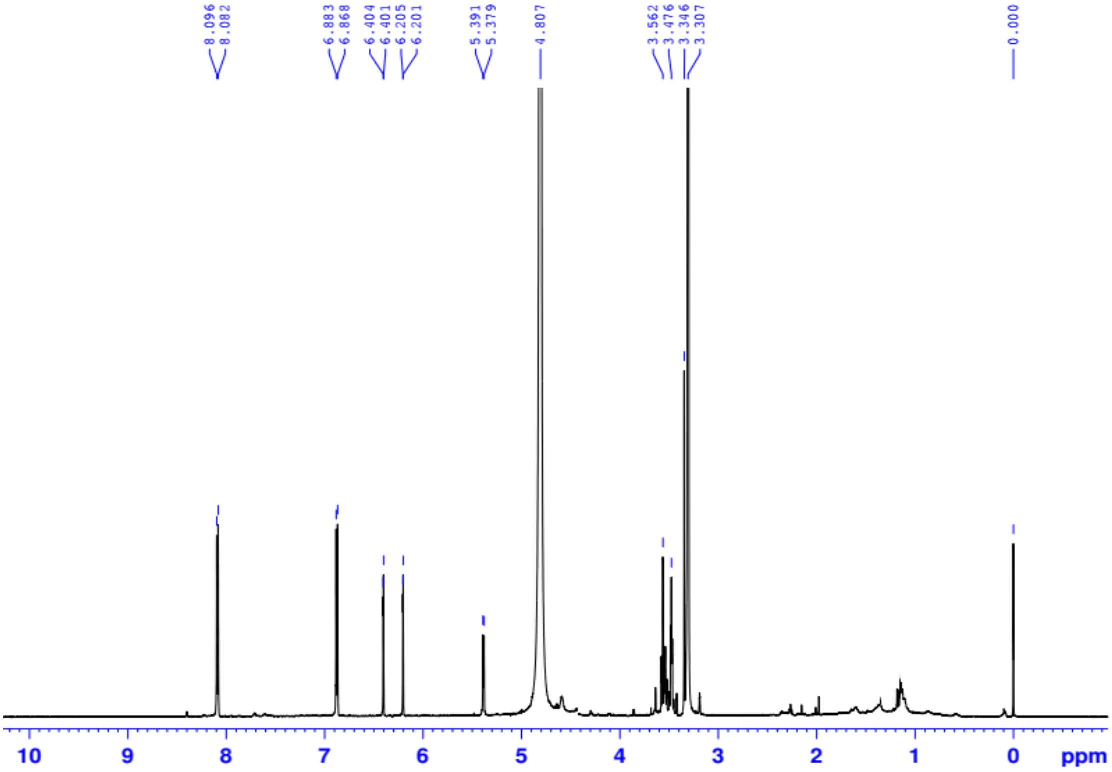

Figure S45. ^{1}H NMR spectrum (600 MHz, CD_3OD) compound 13

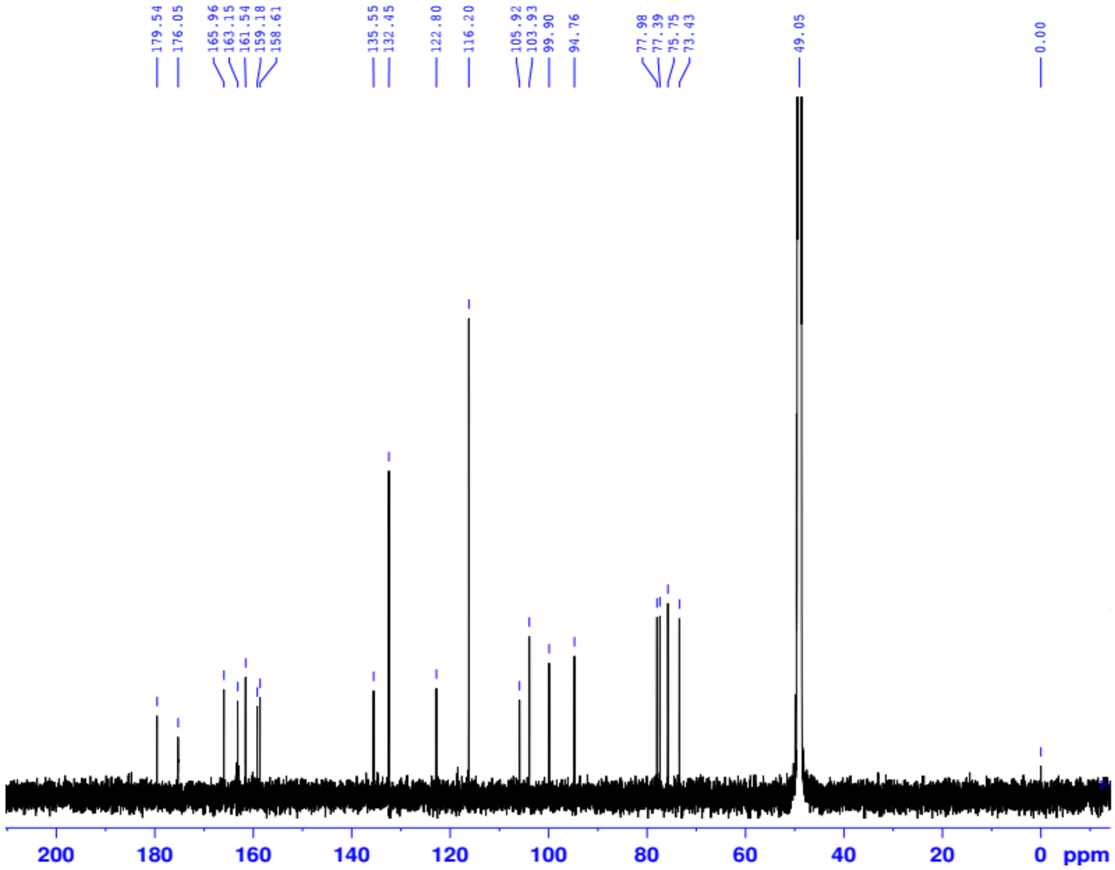

Figure S46. ^{13}C NMR spectrum (150 MHz, CD_3OD) of compound 13

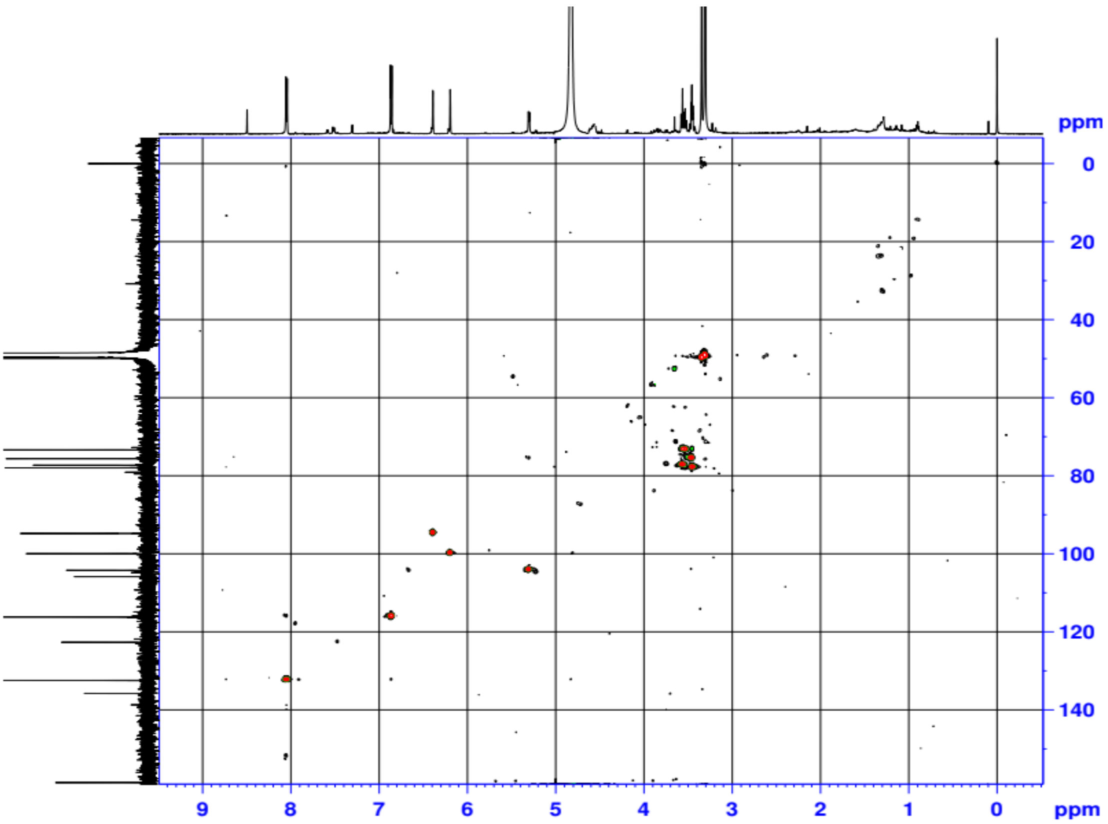

Figure S47. HSQC spectrum (CD_3OD) of compound 13

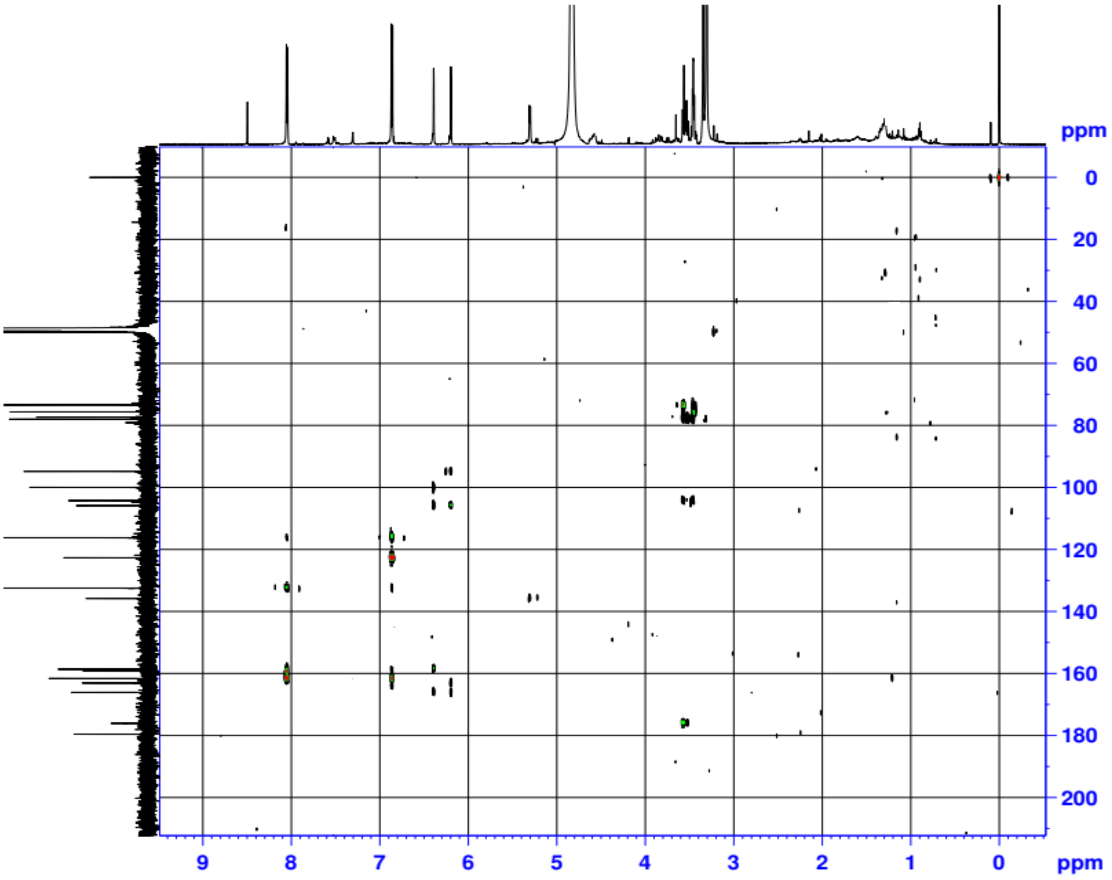

Figure S48. HMBC spectrum (CD3OD) of compound 13

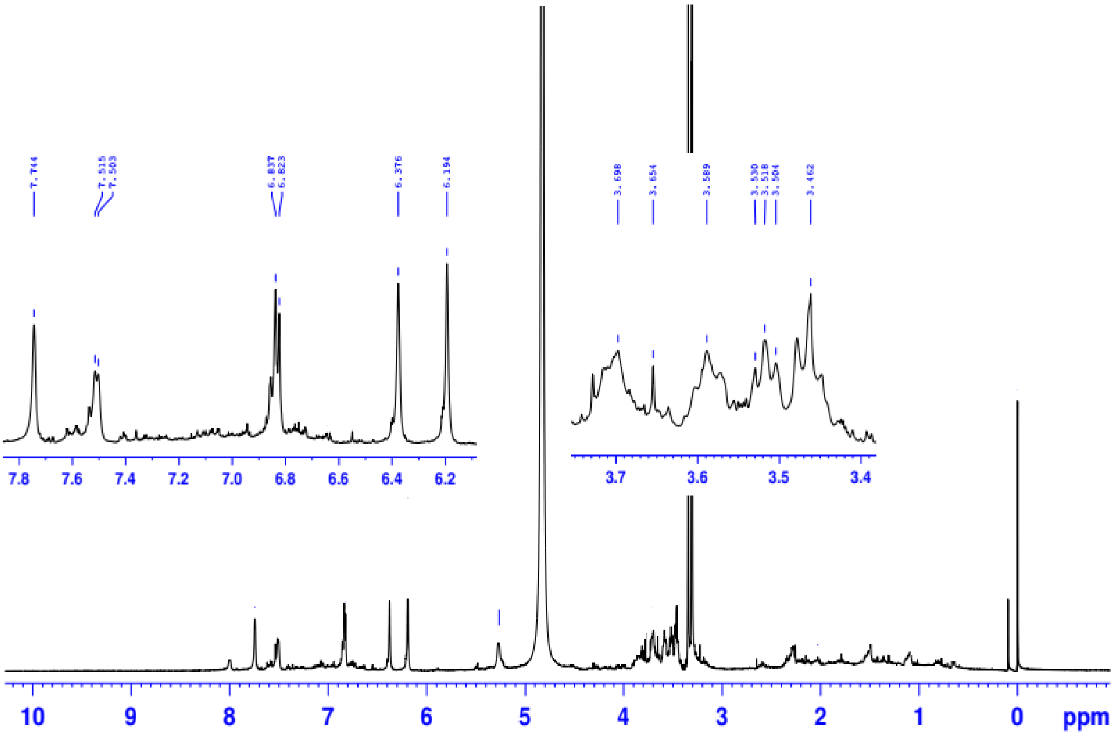

Figure S49. ^{1}H NMR spectrum (600 MHz, CD3OD) of compound 14

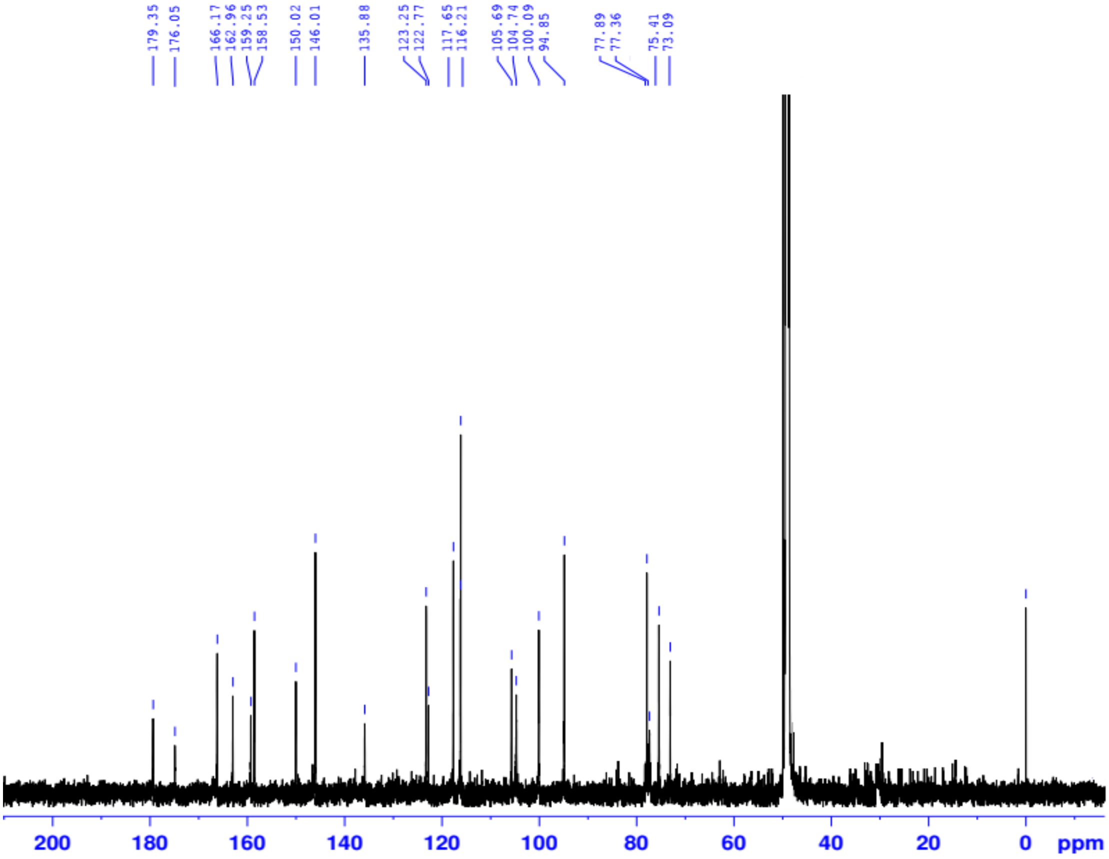

Figure S50. ^{13}C NMR spectrum (150 MHz, CD_3OD) of compound 14

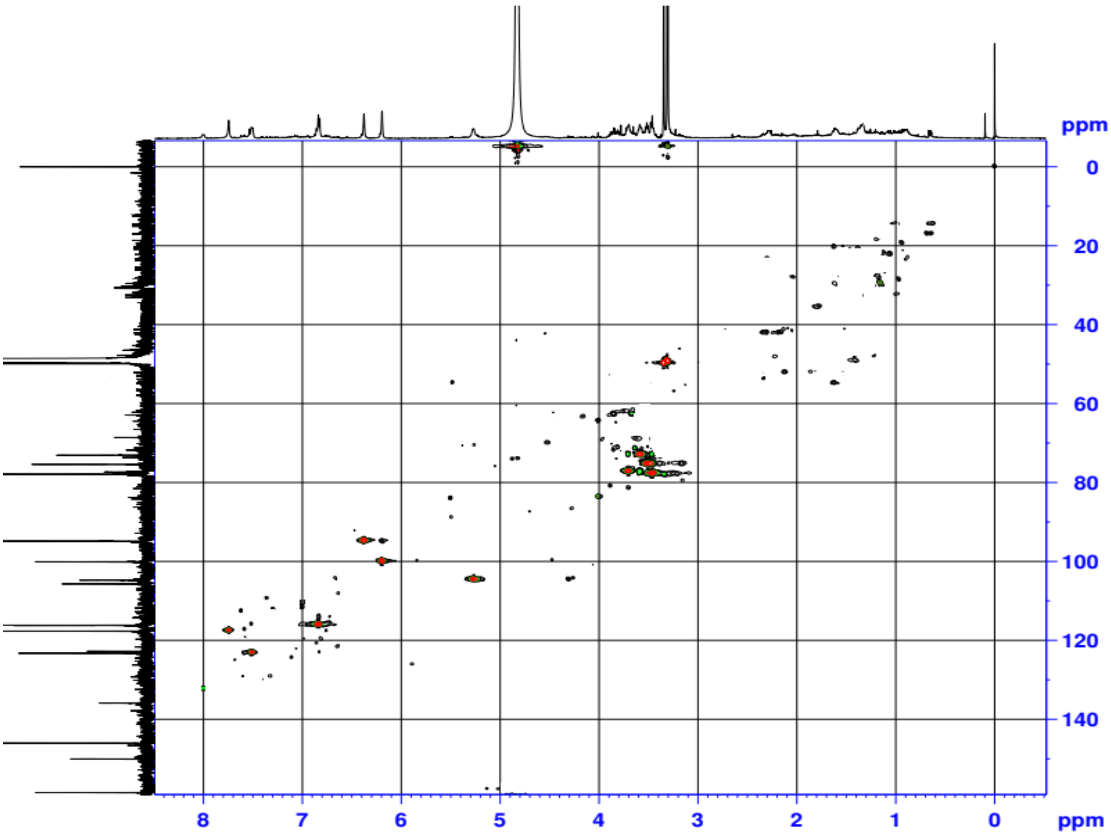

Figure S51. HSQC spectrum (CD_3OD) of compound 14

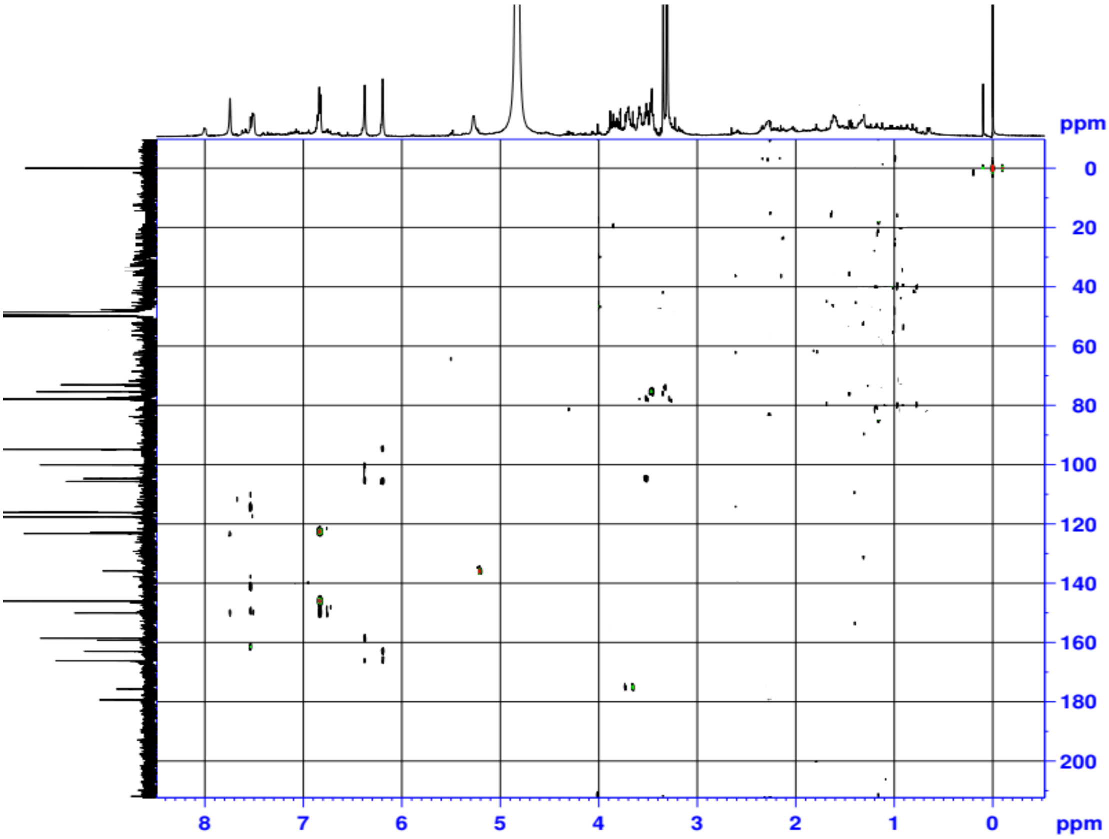

Figure S52. HMBC spectrum (CD_3OD) of compound 14
